# *NLGN3* autism variants have distinct functional impact on synapses and sleep behavior in *Drosophila*

**DOI:** 10.1371/journal.pgen.1012341

**Published:** 2026-10-16

**Authors:** Rebekah E. Townsley, Jonathan C. Andrews, Saurabh Srivastav, Sharayu V. Jangam, Shabab B. Hannan, Oguz Kanca, Paul C. Marcogliese, Shinya Yamamoto, Michael F. Wangler

**Affiliations:** 1 Department of Molecular and Human Genetics, Baylor College of Medicine, Houston, Texas, United States of America; 2 Jan and Dan Duncan Neurological Research Institute, Texas Children’s Hospital, Houston, Texas, United States of America; 3 Department of Neurology, Baylor College of Medicine, Houston, Texas, United States of America; 4 Department of Biochemistry and Medical Genetics, University of Manitoba, Winnipeg, Manitoba, Canada; 5 Children’s Hospital Research Institute of Manitoba, Winnipeg, Manitoba, Canada; 6 Department of Neuroscience, Baylor College of Medicine, Houston, Texas, United States of America; Pennsylvania State University, UNITED STATES OF AMERICA

## Abstract

*Neuroligin-3* (*NLGN3*) was first identified as a X-linked risk gene associated with autism spectrum disorder (ASD). Most reported variants are identified in males who inherited their variants from an unaffected mother, but *de novo* variants have also been identified in both affected heterozygous females and hemizygous males. The p.R451C variant was the initial variant associating *NLGN3* with ASD and has been heavily investigated, yet little is known about the functional consequences of other *NLGN3* variants. To determine the functional consequences of other *NLGN3* variants *in vivo*, we generated transgenic *Drosophila* models corresponding to a *de novo* variant (p.R175W) identified in a heterozygous female proband with ASD, a maternally inherited variant (p.R451C) identified in a hemizygous male with ASD, and another maternally inherited variant identified in a male (p.R597W) associated with sleep disorder and ASD. In *Drosophila*, loss of the fly homolog, *Nlg3,* altered sleep patterns, synaptic architecture, and vesicle dynamics, which were rescued by the expression of the human *NLGN3*^*Ref*^ allele. When comparing the variants, the p.R175W variant and the maternally inherited p.R451C variant altered synapse morphology with minimal effects on vesicle dynamics, and the p.R597W variant altered sleep and vesicle dynamics with minimal impact on synapse morphology. Using overexpression models, human *NLGN3*^*Ref*^ altered sleep patterns and synaptic morphology. Moreover, the p.R175W variant exacerbated sleep phenotypes, and the p.R175W and p.R451C variants exacerbated synapse morphology phenotypes. Together, our findings suggest that the *de novo NLGN3* p.R175W variant identified in a heterozygous female exhibits gain-of-function properties, while maternally inherited variants have context-dependent functional impact. Moreover, some *NLGN3* variants disrupt synaptic development, while other variants alter synaptic function, suggesting that *NLGN3* variants have differential effects. These functional differences may provide insight into the heterogeneity of individuals with ASD.

## Author summary

Autism spectrum disorder (ASD) is a common neurodevelopmental disorder. Variants in the X-linked *Neuroligin-3* (*NLGN3*) gene are associated with ASD, but very few of these changes have been characterized in animal models. Most of these variants affect hemizygous male individuals who maternally inherited their genetic alteration from unaffected heterozygous maternal carriers; however, more rarely affected female individuals may present with a heterozygous variant that was not identified in either of the parents. Here, we utilized the fruit fly model to investigate how three different variants, one being identified in a heterozygous female and two variants identified in hemizygous males, affect the fly’s behavior and synapse development. We identified altered sleep patterns when some *NLGN3* variants are expressed which is consistent with sleep disturbances being highly comorbid with ASD. Additionally, we identified alterations in synapse development and function which is consistent with the role of NLGN3 in synapse formation and maturation. Together, our findings support that NLGN3 is important for regulating the synapse and pathogenic variants are not all equivalent and may fall into distinct classes of functional impact depending on context. This demonstrates the utility of assessing multiple variants simultaneously because each variant may have distinct functional significances.

## Introduction

Autism spectrum disorder (ASD) is a highly heritable complex neurodevelopmental disorder characterized by deficits in social communication and interaction, and restricted, repetitive behaviors [1]. It affects approximately 1 in 31 children in the United States annually, with boys diagnosed 3.4 times more than girls [2]. Additionally, about 74% of individuals with ASD have at least one other co-occurring neurological or psychiatric disorder, including intellectual disability, attention-deficit hyperactivity disorder, anxiety, and sleep disorders [3–6]. Although numerous ASD susceptibility genes have been identified, many converge on pathways regulating neuronal communication and brain development, including neurite outgrowth and synaptic function [7–10].

The *Neuroligin* (*NLGN*) gene family was among the first genes associated with ASD [11]. NLGNs are a family of postsynaptic cell adhesion molecules that regulate synapse formation and maturation with their binding partner, Neurexin [12–15]. Their intracellular domain contains postsynaptic density 95/discs large/zona occluden-1 (PDZ) and gephyrin binding motifs that indirectly recruit GABAergic and glutamatergic receptors to the postsynaptic membrane [16,17]. This signaling is essential for establishing synaptic contacts and maintaining synaptic plasticity, both crucial for proper brain function. Humans have five *NLGN* genes that are preferentially expressed and localized at either the excitatory or inhibitory synapses; however, only *NLGN3* is uniquely localized to both [18–21]. *NLGN3,* located on Xq13.1, was first associated with ASD when a maternally inherited p.R451C variant was identified in two brothers with ASD [11,22]. Since then, its functional impact has been heavily investigated in mouse knock-in and human cell models [23–30]. Although most studies support a gain-of-function (GoF) mechanism, emerging evidence suggests a cell type–dependent effect: some brain regions show GoF while others show loss-of-function (LoF) properties [23,26,27,31]. Beyond the p.R451C variant, at least 23 additional *NLGN3* missense variants have been associated with ASD, most inherited maternally [32,33]. While the p.R451C variant has been well studied, the functional impact of other ASD-associated *NLGN3* variants remains less understood.

Our lab previously used the *Drosophila melanogaster* model organism to screen 79 *de novo* ASD variants in 74 genes and identified that 38% of these variants cause functional changes in *Drosophila* [34]. Using a humanization strategy, we can determine whether variants act in a LoF, GoF, or in a complex context-dependent manner [34–38]. With a more simplified neuronal architecture, the *Drosophila* neuromuscular junction (NMJ) also provides functional insight into genes regulating synapse development and plasticity [39]*. Drosophila* has four *Neuroligin* (*Nlg*) genes; *Drosophila Nlg3* is homologous to human *NLGN3* [40–43]. Furthermore, *Drosophila Nlg* genes are known to alter synaptic development at the NMJ. *Nlg1*, *Nlg2,* and *Nlg4* mutants reduce bouton number while *Nlg3* mutants increase bouton number, and all *Nlg* mutants impair synaptic transmission [40,42–46]. Like its mammalian counterpart, *Nlg3* is also the only member that is localized to both excitatory and inhibitory synapses [47,48]. While loss of *Nlg3* has been characterized in knockout models, we and others have developed transgenic toolkits to rapidly assess the functional impact of human missense variants [40,47]. In this study, we utilized *Drosophila* to investigate whether human NLGN3 can functionally replace the fly protein and assess the functional impact of three ASD-associated *NLGN3* variants: two maternally inherited missense variants and one female *de novo* missense variant. We investigated their impact on *Drosophila* behavior, synapse morphology, and vesicle dynamics through humanization and overexpression strategies. These findings help elucidate the functional impact of *NLGN3* variants and highlight the importance of investigating multiple variants within the same gene as different variants can have differential effects in a context-dependent manner.

## Results

### Selection of variants

*NLGN3* was associated with ASD in 2003 when a maternally inherited missense variant (p.R451C) was observed in two brothers [11]. Since then, at least 23 *NLGN3* missense variants have been identified in individuals with ASD, many of which remain functionally uncharacterized [32,33]. Of these, 5 are *de novo*, 13 are maternally inherited, and 5 are of unknown inheritance. Notably, most *NLGN3* variants affect hemizygous males, and these variants are typically inherited from heterozygous female carriers who are unaffected; however, more rarely, affected females exhibit *de novo* heterozygous variants. Since *NLGN3* is an X-linked gene, random X-inactivation occurs in females allowing heterozygous females to tolerate the variant but leads to disease in hemizygous males. However, we observed *de novo* heterozygous variants in affected females as well, suggesting potential differences in their functional effects.

To assess whether different variants have distinct functional effects, we queried the Simons Foundation Autism Research Initiative (SFARI) and ClinVar databases for ASD-associated *NLGN3* missense variants [32,33]. We identified 13 variants from SFARI and 10 variants from ClinVar that were associated with ASD. We then used *in silico* prediction tools to determine the pathogenicity of the variants and performed a literature review to identify all clinical phenotypes associated with each variant (S1 Table). We removed any variants where the inheritance pattern or biological sex was unknown (S1 Table). To prioritize variants most likely to cause functional changes, we removed variants that were not highly conserved across vertebrate species and where the *in silico* prediction tools suggested “likely benign” (S1 Table and S1 Fig). We also considered whether the variants were associated with other neurological disorders such as sleep disruptions. Intriguingly, two individuals had dual diagnosis where they had one *NLGN3* variant and a second variant in an ASD associated gene (*ASH1L* and *EHMT1*), suggesting a multigenic cause. Both genes are associated with ASD, and additionally *EHMT1* causes Kleefstra Syndrome and *ASH1L* is associated with intellectual disability. Since multigenic causes are difficult to interpret, the *NLGN3* variants identified in these individuals were removed from further analysis (S1 Table). After filtering, we focused on three different categories: *De novo* variants in affected females, maternally inherited variants with sufficient clinical data, and as noted previously we also focused on variants that are observed in individuals with sleep disturbances (S1 Table). We prioritized the top candidate variant for each of the listed categories. We only observed two *de novo* variants in affected females. Although both variants are in regions that are not well conserved across species, we selected the p.R175W variant because it had the highest combined annotation dependent depletion (CADD) score and was more likely to be deleterious while the other variant (p.G406S) is predicted to be benign. Six variants were maternally inherited and had sufficient clinical data. Of these six, we prioritized the p.R451C variant because it had the highest CADD score. This variant has also been heavily investigated in rodent models but not functionally studied in *Drosophila*. Therefore, we were interested in understanding whether this variant could cause functional changes even in an invertebrate species. Lastly, there were two variants identified in individuals with sleep disruptions. Of these two variants, we prioritized the p.R597W variant because *in silico* prediction tools predicted it to be more deleterious than the p.P120L variant. Notably, p.R451C and p.R597W variants have been previously studied *in vivo* or *in vitro,* respectively [11,49,50].

### Bioinformatic evaluation of ASD-associated *NLGN3* variants

The first missense allele that we selected (p.R175W) was identified as a *de novo* variant in a heterozygous female, diagnosed with ASD and intellectual disability (Fig 1A) [51]. The second variant (p.R451C) was maternally inherited in two hemizygous brothers diagnosed with ASD (Fig 1A) [11]. The third variant (p.R597W) was maternally inherited in three hemizygous males all diagnosed with ASD, intellectual disability, and sleep disorders (Fig 1A) [49,50]. Although the last two variants were inherited, none of the maternal carriers were affected. All three variants are localized to the cholinesterase-like (Che-like) domain, the primary extracellular domain of NLGN3, which is predicted to be highly intolerant to change by MetaDome analysis, suggesting a potential hotspot for pathogenic variants (Fig 1B) [52]. This domain interacts with Neurexin to regulate synapse maturation, suggesting that disruptive variants in this domain may alter synaptic function. To assess the pathogenic effect of these three variants, we utilized additional *in silico* prediction tools. *In silico* analysis predicted that the p.R451C and p.R597W variants are likely pathogenic, while the p.R175W variant yielded inconsistent results (S2 Table). We also used MutPred2 to predict specific molecular alterations that may be causative of disease [53]. Mutpred2 predicted distinct molecular mechanisms for the p.R175W variant while the other two variants had similar molecular mechanisms. Specifically, the p.R175W variant is predicted to become more rigid and less regulated, suggesting it may alter its interaction with other binding partners. While the other two variants are predicted to alter the transmembrane, suggesting it may be misfolded or mislocalized (S2 Table). Lastly, all three variants affect highly conserved residues. The p.R451 and p.R597 residues are conserved across all vertebrate and invertebrate species analyzed as well as across all *NLGN* family members; however, the p.R175 residue is only conserved in vertebrates and mildly conserved among *NLGN* paralogs (Figs 1C and S2A). These findings suggest that these residues are critical for the function of *NLGN3*; however, they may have different functional consequences. To study this, we used the *Drosophila melanogaster* model organism.

**Fig 1:**
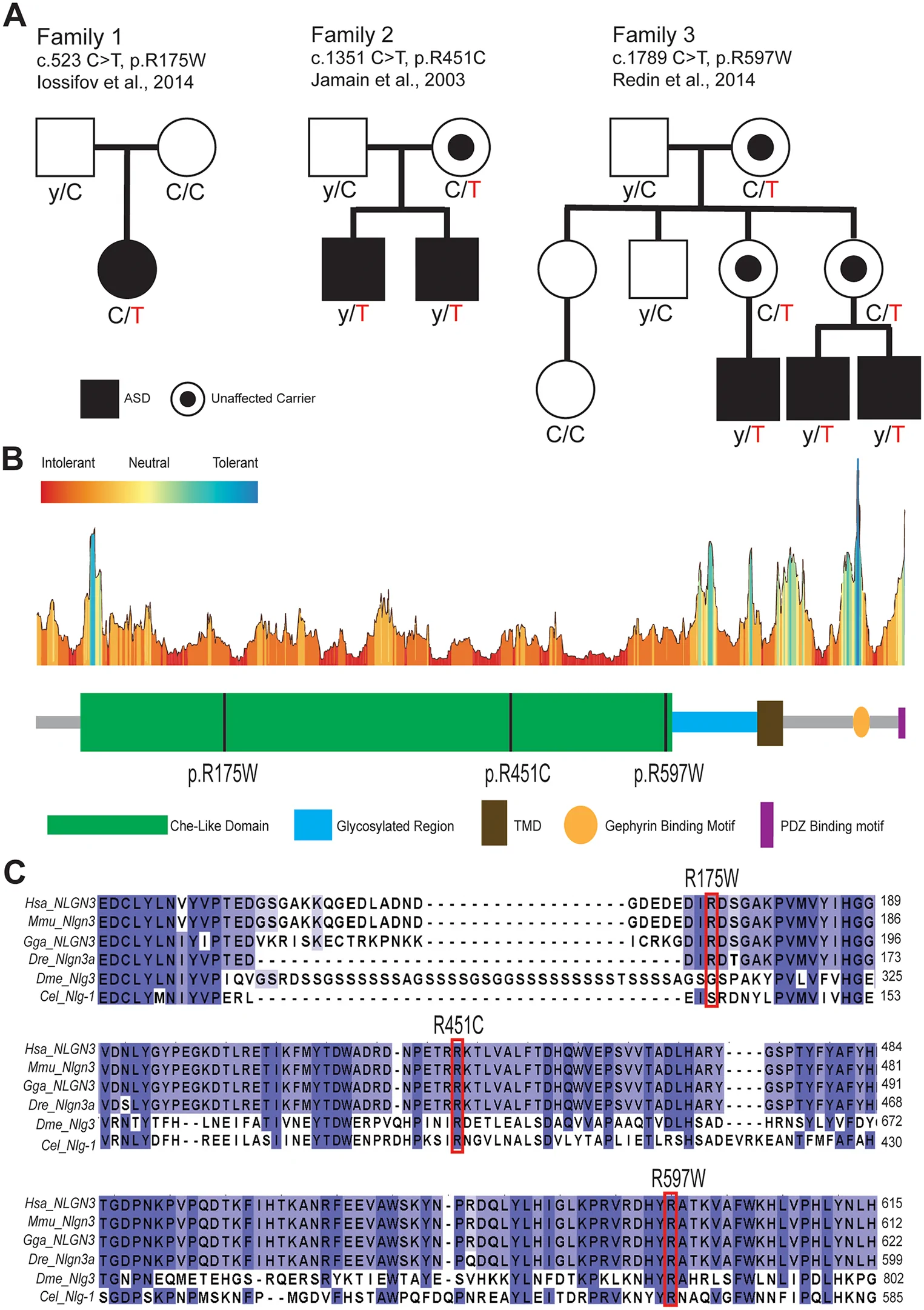
Pedigree and conservation analysis. **A)** Family pedigrees. Open squares or circles represent unaffected males or females, respectively. Closed squares or circles represents affected males or females, respectively. A filled circle inside an open circle represents unaffected female carriers. **B)** Functional domains of NLGN3 and variants’ location using MetaDome analysis. Functional domains of NLGN3 include cholinesterase-like domain (Che-like), glycosylated region, transmembrane domain (TMD), Gephyrin binding motif, and PSD-95/DLG1/ZO-1 binding motif (PDZ motif). **C)** Evolutionary conservation of *NLGN3* variants. Human (NM_018977.4), mouse (Q8BYM5), chicken (A0A8V0Z5J3), zebrafish (D0EVX1), *Drosophila* (Q9VIC6), and *C. elegans* (Q9XTG1) were used.

### *Drosophila Nlg3* is expressed in neurons

*Drosophila* has four *Nlg* family genes: *Nlg1, Nlg2, Nlg3,* and *Nlg4* [40–43]. *Nlg3* shows the highest homology to human *NLGN3* (DIOPT score = 12, 32% identity and 46% similarity) [54]. To study the functional impact of patient-derived variants, we used a previously generated *Nlg3*-specific trojan GAL4 (TG4) allele [34,55]. The *Nlg3*^*TG4*^ allele contains a splice donor, T2A ribosomal skipping sequence, GAL4 transcriptional activator, and a polyA signal, and it is inserted between exons 3 and 4 (Fig 2A) [55]. This insertion truncates the endogenous protein and expresses a GAL4 transcriptional activator in the same spatial and temporal manner. The GAL4 protein can drive the transcription of cDNAs that are cloned downstream of an upstream activation sequence (UAS). This allele can be used to detect the expression pattern of fly *Nlg3* and express human *NLGN3* cDNA in the expression domain of *Nlg3*. *NLGN3* is primarily expressed in neurons but has been identified in astrocytes and plays a role in neuron-glia interaction [56]. To assess the lineage trace expression pattern of fly *Nlg3*, we crossed *Nlg3*^*TG4*^ with a G-Trace reporter and stained adult brains with neuronal marker Elav and glial marker Repo [57]. Similar to humans, *Nlg3* is primarily expressed in neurons and sparsely expressed in glia throughout development (Figs 2B–2I and S2B–S2G).

**Fig 2:**
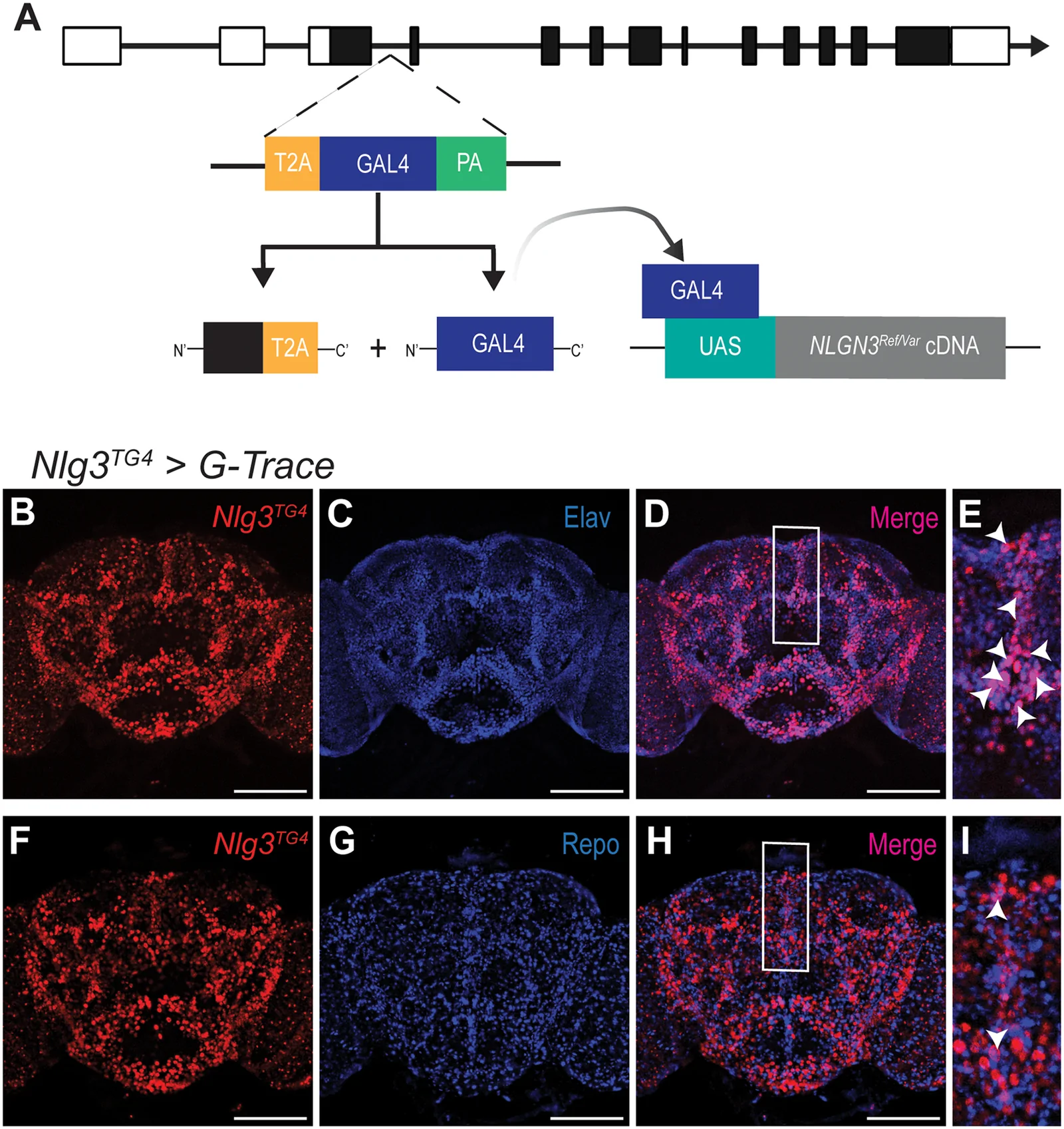
Humanization strategy enables spatiotemporal control of *Nlg3* expression. **A)** The *Nlg3* specific trojan GAL4 (*Nlg3*^*TG4*^) allele truncates the fly protein but produces a GAL4 transcriptional activator in the same spatiotemporal pattern as the endogenous fly gene. The GAL4 protein can drive the expression of UAS-*NLGN3* constructs or a fluorescent reporter. **B, F)** *Nlg3*^*TG4*^ driving a G-trace reporter line was used to identify the current expression pattern of *Drosophila Nlg3*. **C)** anti-Elav staining labels neurons. **D, E)** Merging the expression of *Nlg3* and Elav positive cells identifies that *Nlg3* is expressed in neurons. **G)** anti-Repo staining labels glial cells. **H, I)** Merging *Nlg3* and Repo positive cells shows some overlap between *Nlg3* and glia suggesting *Nlg3* is sparsely expressed in glia. White arrowheads indicate colocalization. Scale bar: 100 μm.

### *NLGN3* alters locomotion and sleep behaviors

Although the *Nlg3*^*TG4*^ allele is predicted to function as a strong LoF allele, it has not been fully characterized. To confirm this, we first quantified the total mRNA expression in adult heads and compared it to a known null allele (*Nlg3*^*Null*^) [47]. This previously generated *Nlg3*^*Null*^ is a small deficiency allele that knocks out six genes including *Nlg3*. The remaining five genes encode carboxylesterases (α-Est1, α-Est2, α-Est3, α-Est4, and CG45545). While the knockdown of all five genes is viable, their role in fly behavior is poorly understood, yet we expect the trans-heterozygous loss of these genes in a *Nlg3*^*TG4/Null*^ background to have minimal impact on fly behavior. Using primers downstream of the insertion site, we observed that the *Nlg3*^*TG4/Null*^ flies have 87% and *Nlg3*^*Null/Null*^ flies have 95% decrease in *Nlg3* mRNA levels; however, both mutants are viable (Fig 3A). To determine if loss of *Nlg3* leads to neuronal phenotypes such as a shortened lifespan, we recorded longevity in *Nlg3* mutants. Interestingly, control and *Nlg3*^*Null/Null*^ flies had a median survival rate of 46 days, but the *Nlg3*^*TG4/Null*^ flies had a median survival rate of 57 days (Fig 3B). This could be attributed to the *Nlg3*^*TG4*^ being a hypomorph rather than an amorph or differences in genetic backgrounds.

**Fig 3:**
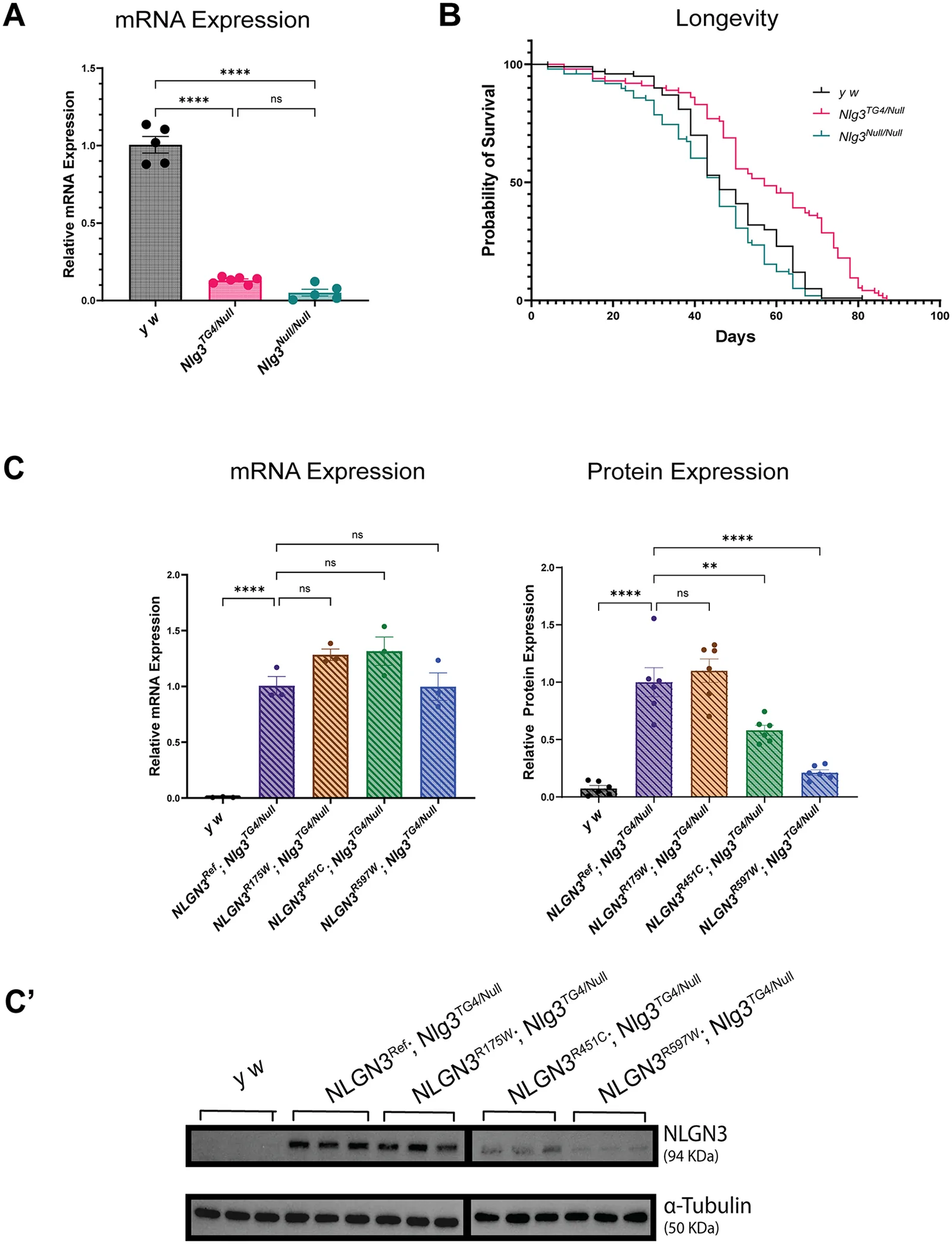
*NLGN3* variants alter protein expression. **A)** Relative mRNA expression in *Nlg3*^*TG4/Null*^ and *Nlg3*^*Null/Null*^ flies (5-6 biological replicates, n = 20 heads per biological replicate, 3 technical replicates; One-way ANOVA with Sidak’s multiple comparison test, ns *P* > 0.05, ***P ≤ 0.001). **B)** Survival curves of *y*^*1*^ w^*^ (controls), *Nlg3*^*TG4/Null*^ and *Nlg3*^*Null/Null*^ flies. *Nlg3*^*TG4/Null*^ mutants have increased lifespan compared to controls (χ^2^ = 26) while *Nlg3*^*Null/Null*^ mutants have reduced lifespan compared to controls (χ^2^ = 5.18) (n = 100 adult flies per genotype, Mantel Cox Logrank test). **C)** Relative mRNA expression in *y*^*1*^ w^*^, NLGN3^*Ref*^*; Nlg3*^*TG4/Null*^*, NLGN3*^*R175W*^*; Nlg3*^*TG4/Null*^, *NLGN3*^*R451C*^*; Nlg3*^*TG4/Null*^, and *NLGN3*^*R597W*^*; Nlg3*^*TG4/Null*^ flies (3 biological replicates, n = 20 heads per biological replicate, 3 technical replicates; One-way ANOVA with Sidak’s multiple comparison test; ns *P >* 0.05, **** *P* ≤ 0.0001). Quantification of NLGN3 band intensity normalized to α-Tubulin band intensity (n = 6, One way ANOVA with Sidak’s multiple comparison test; ns *P*  > 0.05, ** *P* ≤ 0.001, **** *P* ≤ 0.0001). **C’)** Representative western blot for *y*^*1*^ w^*^, NLGN3^*Ref*^*; Nlg3*^*TG4/Null*^*, NLGN3*^*R175W*^*; Nlg3*^*TG4/Null*^*, NLGN3*^*R451C*^*; Nlg3*^*TG4/Null*^, and *NLGN3*^*R597W*^*; Nlg3*^*TG4/Null*^ whole head lysate probed for NLGN3 and α-Tubulin.

We next expressed human *NLGN3* reference and variant constructs in a *Nlg3*^*TG4/Null*^ background to determine the functional consequences of these different variants. To ensure human NLGN3 proteins are being expressed, we first measured protein levels. We injected the cDNA into the same docking site; therefore, we would expect similar mRNA expression levels across both the reference and variant alleles. Designing primers specifically targeting human *NLGN3*, we did not detect any human *NLGN3* mRNA expression in the y^*^ *w*^*1*^ flies; however, our primers were able to detect human *NLGN3* mRNA in all four rescue constructs. As expected, we did not observe statistical differences in mRNA expression between our *NLGN3* rescue constructs (Fig 3C). To identify if protein expression is altered, we measured the relative protein expression of human NLGN3. As expected, we did not detect a band for y* w^*1*^ flies; however, a band was detected for all *NLGN3* rescue constructs, suggesting that human NLGN3 proteins are being expressed in our fly model. The *NLGN3*^*R175W*^ variant had similar protein levels compared to the *NLGN3*^*Ref*^, suggesting that this variant is likely stable. However, the *NLGN3*^*R451C*^ variant had a 42% reduction, and the *NLGN3*^*R597W*^ variant had a 79% reduction in protein levels (Fig 3C). This is consistent with previous *in vitro* findings, suggesting that the *NLGN3*^*R451C*^ and *NLGN3*^*R597W*^ variants may be retained in the endoplasmic reticulum and degraded [23,49].

To further assess if the *Nlg3*^*TG4*^ allele shows phenotypic similarities to the *Nlg3*^*Null*^ alleles and whether human *NLGN3* reference or variant alleles can rescue, we next assessed locomotor activity. Loss of *Nlg3* is known to cause deficits in locomotion [40,47]. To measure motor activity, we recorded basal activity of *Nlg3*^*TG4/Null*^ and *Nlg3*^*Null/Null*^ flies using the *Drosophila* activity monitor [58]. Consistent with previous literature, we observed a significant decrease in male and female total activity in both of these allelic combinations (Fig 4A). To determine if this reduction in activity is the result of a motor deficit, we next measured the activity of the animals when they were in a “wake” state. Consistent with previous literature, we observed that males exhibited a reduction in their activity/waking minute, suggesting a motor deficit; however, females exhibited an increase in activity/waking minute, suggesting that these animals are hyperactive when they are in a “wake” state (S3A-S3B Fig). Since the *Nlg3*^*TG4*^ allele is mirroring the behavior of the *Nlg3*^*Null*^ allele, we hypothesize that the *Nlg3*^*TG4*^ is likely functioning as a LoF allele [40,47]. The expression of the human *NLGN3*^*Ref*^, *NLGN3*^*R451C*^*,* and *NLGN3*^*R597W*^ variants in a *Nlg3*^*TG4/Null*^ background in males failed to rescue this activity defect (Fig 4A). These findings were recapitulated when we measured the amount of time the fly spent moving within a test chamber in a 30-minute period (S3B Fig). However, the expression of *NLGN3*^*R175W*^ variant appears to partially rescue locomotor defect observed over a 7-day period but fails to rescue acute locomotion observed over a 30-minute timeframe (Figs 4A and S3B). In females, the expression of the *NLGN3* reference or variant does not rescue total activity; however, activity/waking minute was rescued and no defects in locomotion over a 30-minute timeframe was observed (Figs 4A and S3A). Overall, this suggests that males may exhibit a motor defect that is not observed in females. Since human *NLGN3* failed to rescue locomotion, we next tested if fly *Nlg3*^*WT*^ could rescue locomotion to determine if this effect was due to the function not being conserved in human *NLGN3*. Similarly, the expression of *Nlg3*^*WT*^ in a *Nlg3*^*TG4/Null*^ failed to rescue locomotor deficits (S3 Fig). Together these results suggest that either the *Nlg3*^*TG4*^ allele has a second site hit or the expression level of the UAS may be suboptimal, making it difficult to identify if restoring either human or fly *Nlg3* can rescue locomotion in males.

**Fig 4:**
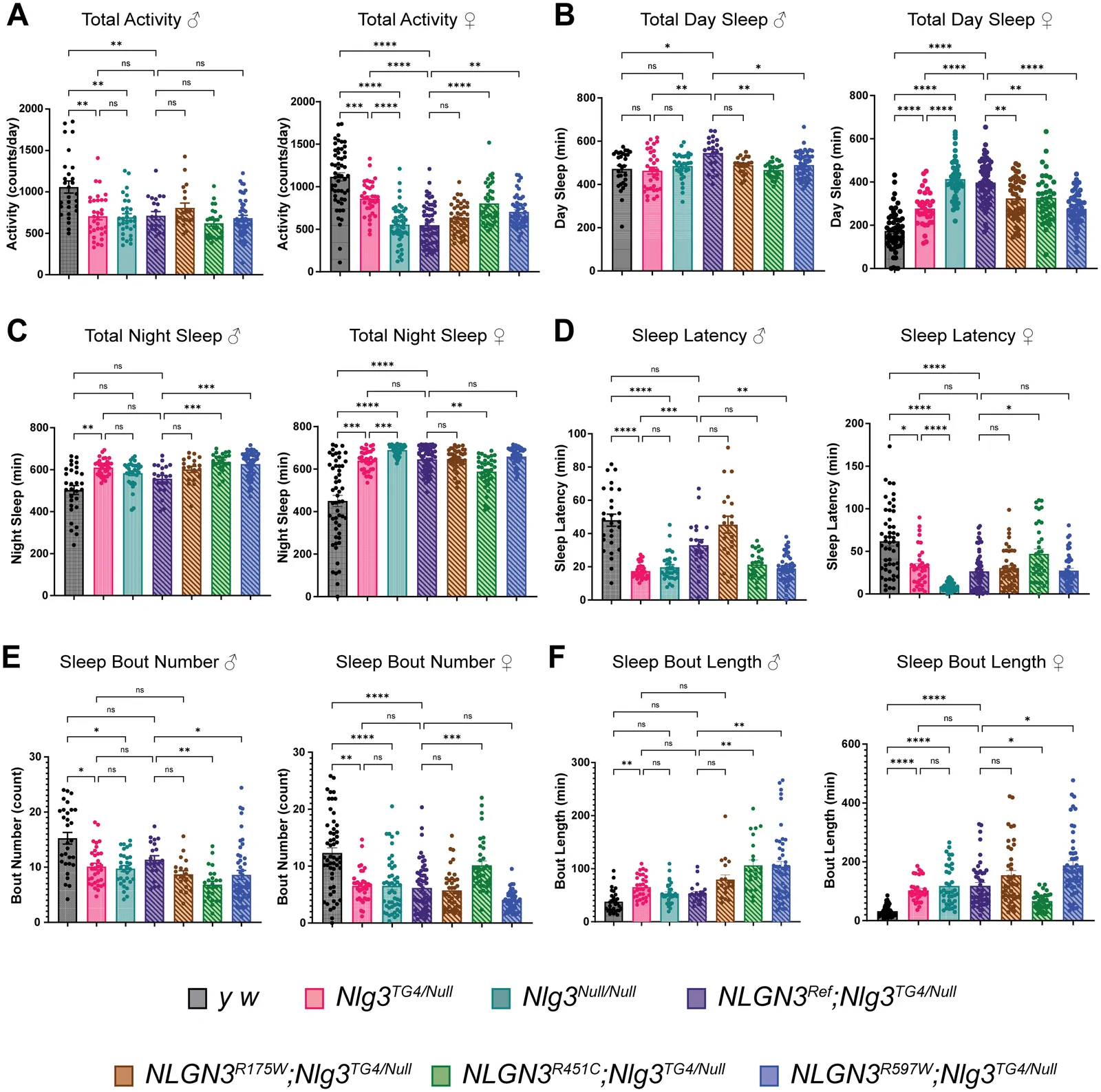
*NLGN3* variants have altered sleep patterns. **A)** Quantification of total locomotor activity in males and females (males: n ≥ 19 flies, females: n ≥ 36 flies; Welch’s ANOVA with Dunnett’s T3 multiple comparison test, ns *P* > 0.05, ** *P* ≤ 0.01, *** *P* ≤ 0.001, **** *P* ≤ 0.0001). **B)** Quantification of sleep duration during lights on in males and females (males: n ≥ 19 flies; Kruskal Wallis with Dunn’s multiple comparison test, females: n ≥ 36 flies; One-way ANOVA with Sidak’s multiple comparison test). **C)** Quantification of sleep duration during lights off in males and females (males: n ≥ 19 flies, females: n ≥ 36 flies). **D)** Quantification of sleep latency in males and females (males: n ≥ 19 flies, females: n ≥ 36 flies). **E)** Quantification of sleep bout number during lights off in males and females (males: n ≥ 19 flies, females: n ≥ 36 flies). **F)** Quantification of sleep bout length during lights off in males and females (males: n ≥ 19 flies, females: n ≥ 36 flies). Kruskal Wallis with Dunn’s multiple comparison test was used for C-F; ns *P* > 0.05, * *P* ≤ 0.05 ** *P* ≤ 0.01, *** *P* ≤ 0.001, **** *P* ≤ 0.0001).

Since all three variants were identified in individuals with ASD, we additionally measured grooming and courtship phenotypes as readouts for repetitive behaviors and social communication. In males, we observed that the loss of *Nlg3* did not alter grooming or courtship behaviors, whereas the expression of human *NLGN3*^*Ref*^ increased grooming behaviors and the expression of the variants significantly decreased grooming and increased courtship behaviors (S3B Fig). Since *Nlg3* mutants did not exhibit grooming or courtship behaviors, the altered behavior observed in human *NLGN3* alleles is likely an artifact of overexpression. In females, only grooming behaviors were recorded because female courtship behaviors, such as kicking, ovipositor extrusion, or opening the vaginal plate, are subtle and often below the detectable threshold for our tracking system. Loss of *Nlg3* in females increased grooming behaviors; however, the expression of *Nlg3*^*WT*^*, NLGN3*^*Ref*^*, NLGN3*^*R175W*^*,* and *NLGN3*^*R451C*^ in a *Nlg3*^*TG4/Null*^ background did not rescue this defect. While the expression of the *NLGN3*^*R597W*^ variant in a *Nlg3*^*TG4/Null*^ background nominally improved grooming behaviors, although not significantly (S3A Fig). Overall, this suggests that male and females exhibit sexually dimorphic behaviors.

Since ASD and sleep are highly comorbid and one of the variants was associated with sleep disorders in multiple individuals, we next assessed whether *Nlg3* is important for sleep function [49,50]. Previous rodent models have implicated *NLGN3* in sleep disruptions, particularly alterations in the sleep/wake cycle [59,60]. We found that loss of *Nlg3* in males does not affect the duration of daytime sleep, but nighttime sleep was increased. Interestingly, the expression of the *NLGN3*^*Ref*^ in a *Nlg3*^*TG4/Null*^ background increased total daytime sleep while nominally improving, although not significant, nighttime sleep defects. The expression of all three variants in a *Nlg3*^*TG4/Null*^ background failed to rescue the duration of nighttime sleep (Fig 4B–4C). In addition to the duration of sleep, we also evaluated sleep latency and sleep consolidation. Sleep latency refers to the amount of time it takes for the animal to enter their first sleep episode after lights off, and sleep consolidation is a measure of how continuous the sleep episodes are and is composed of the number of sleep episodes and the length of the sleep episodes. Loss of *Nlg3* also drastically reduced sleep latency and increased sleep consolidation while expression of *NLGN3*^*Ref*^ in a *Nlg3*^*TG4/Null*^ background was able to rescue sleep latency defects and improve sleep consolidation defects. The expression of the *NLGN3*^*R451C*^ and *NLGN3*^*R597W*^ variants in a *Nlg3*^*TG4/Null*^ background failed to rescue sleep latency and sleep consolidation defects while the expression of the *NLGN3*^*R175W*^ variant was able to fully rescue sleep latency but failed to rescue sleep consolidation defects (Fig 4D–4F). In females, loss of *Nlg3* increased both day and nighttime sleep duration. The expression of human *NLGN3* failed to rescue duration of sleep during the day, yet *NLGN3*^*R451C*^ was able to nominally improve, though not significantly, nighttime sleep duration (Fig 4B–4C). The lack of apparent motor deficit coupled with an increase in sleep duration suggests that female mutants spend more time sleeping. Consistent with males, loss of *Nlg3* in females resulted in decreased sleep latency and increased sleep consolidation. The expression of human *NLGN3*^*Ref*^*, NLGN3*^*R175W*^*,* and *NLGN3*^*R597W*^ in a *Nlg3*^*TG4/Null*^ background failed to rescue sleep latency and sleep consolidation defects; however, the expression of *NLGN3*^*R451C*^ in a *Nlg3*^*TG4/Null*^ background was able to nominally improve both sleep latency and sleep bout number (Fig 4D–4F). Overall, this suggests that loss of *Nlg3* alters sleep behaviors in males and females which can be rescued by human *NLGN3* in a differential manner.

We next overexpressed each of our alleles pan-neuronally to determine if the overexpression of *NLGN3* in neurons causes behavioral deficits. The overexpression of *NLGN3*^*Ref*^ using the neuronal driver *Elav*-GAL4 in males resulted in a decrease in total activity, but the activity/waking minute was not significant, suggesting that motor function is not impaired in these animals (S4A-S4B Fig). Furthermore, overexpressing *NLGN3*^*Ref*^ using the neuronal driver *Elav*-GAL4 increased the duration of day and night sleep, decreased sleep latency and the number of sleep bouts, and increased the length of each sleep bout. However, overexpressing the *NLGN3*^*R175W*^ variant with *Elav*-GAL4 driver further exacerbated many of these phenotypes (S4 Fig). Together, our findings suggest that overexpressing *NLGN3* causes behavioral deficits and the expression of the *NLGN3* variants can further exacerbate these behavioral phenotypes in *Drosophila*.

### *NLGN3* alters synaptic morphology

Since *NLGN3* regulates synapse formation and maturation in multiple species, we hypothesized that *NLGN3* variants may affect synapse morphology and activity, driving these behavioral phenotypes in individuals with ASD. To evaluate synaptic architecture, we first used the third instar *Drosophila* larval NMJ, which is primarily glutamatergic and has large, easily visualized boutons [39]. Loss of *Nlg3* is known to increase the number of type 1B boutons and reduce bouton size while branch number remains unchanged [40]. Co-staining the NMJ with horse radish peroxidase (HRP), a neuronal membrane marker, and discs large (DLG), a postsynaptic subsynaptic reticulum marker, we recapitulated these findings with our LoF mutants (Fig 5A–5C). The expression of *NLGN3*^*Ref*^ and *NLGN3*^*R597W*^ in a *Nlg3*^*TG4/Null*^ background rescued this bouton overgrowth phenotype whereas *NLGN3*^*R175W*^ and *NLGN3*^*R451C*^ variants showed persistent bouton overgrowth, similar to the LoF mutants, suggesting that these variants are unable to rescue bouton growth (Fig 5A–5H). Consistent with previous findings, NMJ branch number was unchanged (S5A Fig). Although *NLGN3*^*Ref*^ rescued bouton number, bouton size remained reduced, indicating that some functions of *Drosophila Nlg3* cannot be rescued by *NLGN3* (Fig 5I). To determine if this deficit is due to a biological difference between fly *Nlg3* and human *NLGN3*, we next quantified bouton morphology in *Nlg3*^*WT*^ animals. The expression of *Nlg3*^*WT*^ in a *Nlg3*^*TG4/Null*^ background rescued bouton number but failed to rescue bouton size further indicating that our *NLGN3*^*Ref*^ allele is behaving similarly to the endogenous fly *Nlg3*^*WT*^ allele (S5B–S5C Fig).

**Fig 5:**
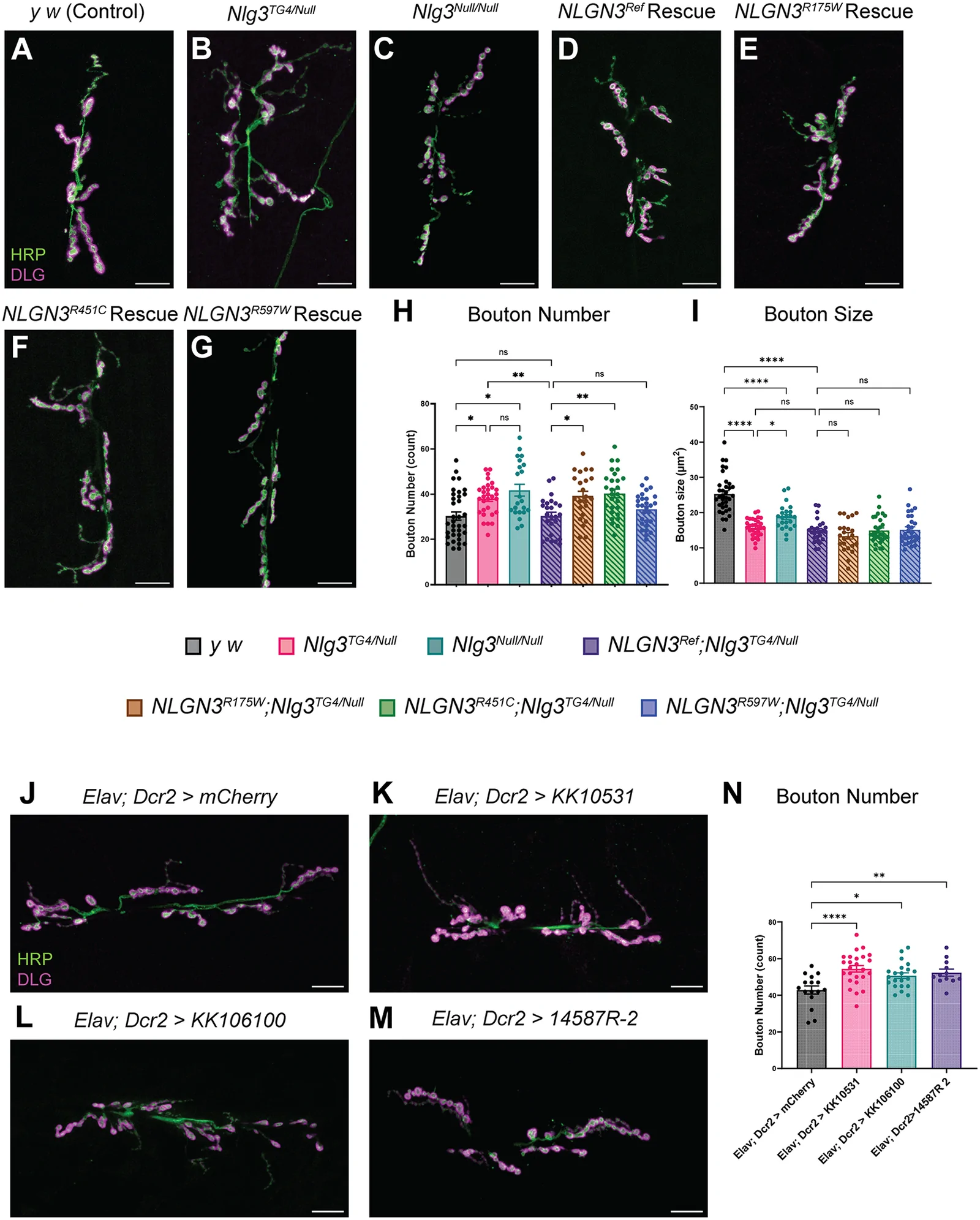
*NLGN3* variants alter synaptic architecture at the neuromuscular junction. **A-G)** Representative images of type 1B boutons at muscle 6/7 of abdominal segment A4 from *y*^*1*^ w^*^, Nlg3^*TG4/Null*^*, Nlg3*^*Null/Null*^*, NLGN3*^*Ref*^*; Nlg3*^*TG4/Null*^*, NLGN3*^*R175W*^*; Nlg3*^*TG4/Null*^*, NLGN3*^*R451C*^*; Nlg3*^*TG4/Null*^, and *NLGN3*^*R597W*^*; Nlg3*^*TG4/Null*^ larvae. Neuromuscular junctions (NMJ) were co-stained with anti-HRP, a pan neuronal marker, and anti-DLG, a postsynaptic marker. **H)** Quantification of the number of boutons at each NMJ (n ≥ 22 larvae/genotype, Welch’s ANOVA with Dunnett’s T3 multiple comparison test; ns *P* > 0.05, * *P* ≤ 0.05, ** *P* ≤ 0.0001. **I)** Quantification of bouton size (n ≥ 22 larvae/ genotype, Welch’s ANOVA with Dunnett’s T3 multiple comparison test; ns *P* > 0.05, * *P* ≤ 0.05, **** *P* ≤ 0.0001). **J-M)** Representative images of type 1B boutons in *Nlg3* knockdown models (*LacZ, KK10531, KK106100, 14587R-2*) using a neuronal driver (Elav-GAL4). NMJs were co-stained with anti-HRP and anti-DLG. **N)** Quantification of bouton number in *Nlg3* knockdown models (n ≥ 12 larvae, One-way ANOVA with Sidak’s multiple comparison test; ns *P* > 0.05, * *P* ≤ 0.05, ** *P* ≤ 0.01, **** P ≤ 0.0001. Scale bar: 20 μm.

*NLGN3* is primarily expressed in neurons; however, some evidence suggests fly *Nlg3* may be expressed in low levels at the larval muscle wall [40]. However, immunofluorescence did not detect *Nlg3* expression at the muscle wall. Thus, it remains unclear which tissue is relevant for bouton growth phenotype. To assess tissue-specific effects, we used overexpression and knockdown models. Overexpressing *NLGN3*^*Ref*^ and *NLGN3*^*R597W*^ with *Nlg3*^*TG4*^ driver in *Nlg3*^*TG4/+*^ larvae did not alter bouton number; however, overexpressing *NLGN3*^*R175W*^ and *NLGN3*^*R451C*^ variants increased bouton number (S6A-S6B Fig). Similar phenotypes were observed when overexpressing human *NLGN3* cDNAs with neuronal driver, *Elav*-GAL4, and motor neuron driver, D42-GAL4 (S6C-S6F Fig). In contrast, muscle specific overexpression (*Mef2*-GAL4) reduced bouton number even in *NLGN3*^*Ref*^ expressing animals (S6G-S6H Fig). Using knockdown models, we identified that the neuronal knockdown of *Nlg3* using *Elav*-GAL4 increased bouton number in three independent RNAi lines, resembling LoF mutants (Fig 5J–5N). This suggests that the loss of *Nlg3* in neurons is sufficient to cause bouton overgrowth and the expression of *NLGN3* variants in the neurons is likely causing the phenotypes we observed.

Since *Drosophila* undergoes extensive neuronal remodeling during pupation, larval and adult neurons are largely distinct populations. We, therefore, assessed if morphological defects observed in larvae could also be observed in adult flies. To ensure the same age as our behavioral and sleep experiments, we examined NMJ morphology in 7-day-old flies. This age corresponds to a fully mature adult fly where neurogenesis and remodeling should be largely complete. Staining the abdominal body wall with cysteine string protein (CSP), a presynaptic marker, we observed an increase in the number of boutons in *Nlg3* mutants (S7A-S7C Fig). The expression of *NLGN3*^*Ref*^ and *NLGN3*^*R597W*^ in a *Nlg3*^*TG4/Null*^ background was able to rescue this bouton phenotype (S7D and S7G Fig). However, similar to the larval NMJ, the expression of the *NLGN3*^*R175W*^ and *NLGN3*^*R451C*^ variants both failed to rescue bouton overgrowth (S7E-S7H Fig). These results indicate that *Nlg3* in *Drosophila* regulates bouton growth throughout development and some *NLGN3* variants impair bouton regulation.

### *NLGN3* variants alter vesicle dynamics

To further examine NLGN3’s role in synapse maturation, we next assessed the morphology of the active zone protein, Bruchpilot (BRP). Co-staining the third instar larval NMJ with BRP and HRP, revealed a significant increase in the number of BRP puncta in the LoF mutants when normalized to the bouton size (Fig 6A–6C). The expression of the *NLGN3*^*Ref*^ in a *Nlg3*^*TG4/Null*^ background was able to fully rescue this phenotype (Fig 6D). Similar to the overgrown bouton phenotype observed earlier, we saw an increase in BRP puncta in animals rescued with either *NLGN3*^*R175W*^ or *NLGN3*^*R451C*^ variants (Fig 6E–6F). However, the *NLGN3*^*R597W*^ variant did not disrupt BRP puncta (Fig 6G–6H). The Che-like domain is important for interacting with Neurexin; therefore, variants (p.R175W and p.R451C) within this hotspot may impair synapse development by disrupting transsynaptic interactions. Together, *NLGN3* seems to be important not only for facilitating synaptic growth but also for active zone regulation.

**Fig 6:**
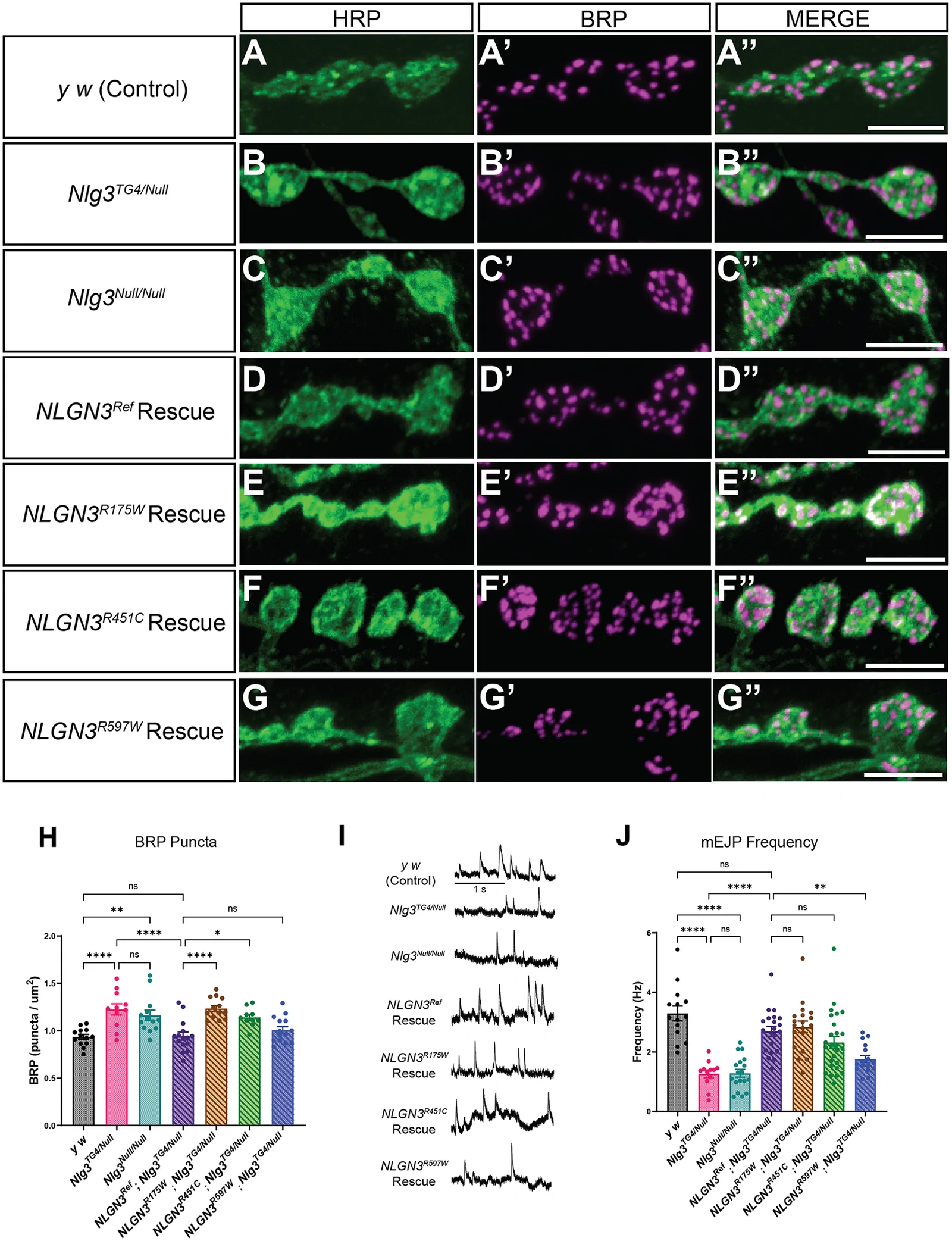
*NLGN3* variants impair vesicle dynamics. **A-G)** Representative images of type 1B boutons at muscle 6/7 of abdominal segment A4 from *y*^*1*^ w^*^, Nlg3^*TG4/Null*^*, Nlg3*^*Null/Null*^*, NLGN3*^*Ref*^*; Nlg3*^*TG4/Null*^*, NLGN3*^*R175W*^*; Nlg3*^*TG4/Null*^*, NLGN3*^*R451C*^*; Nlg3*^*TG4/Null*^, and *NLGN3*^*R597W*^*; Nlg3*^*TG4/Null*^ larvae. Boutons were co-stained with HRP, a pan-neuronal marker, and BRP, an active zone marker. Scale bar: 5 μm. **H)** Quantification of the number of BRP puncta normalized to the size of each bouton (N ≥ 10 larvae/genotype, n = 6 boutons/larvae). One-way ANOVA with Sidak’s multiple comparison test; ns *P* > 0.05, * *P* ≤ 0.05, ** *P* ≤ 0.01, **** *P* ≤ 0.0001. **I)** Representative miniature excitatory junction potentials (mEJP) traces at muscle 6 of abdominal segments A3 and A4 from *y*^*1*^ w^*^, Nlg3^*TG4/Null*^*, Nlg3*^*Null/Null*^*, NLGN3*^*Ref*^*; Nlg3*^*TG4/Null*^*, NLGN3*^*R175W*^*; Nlg3*^*TG4/Null*^*, NLGN3*^*R451C*^*; Nlg3*^*TG4/Null*^, and *NLGN3*^*R597W*^*; Nlg3*^*TG4/Null*^ larvae. Each spike corresponds to one mEJP event. Scale bar: 1 second. **J)** Quantification of the frequency of mEJP (n ≥ 12). Welch’s ANOVA with Dunnett T3 multiple comparison test; ns *P* > 0.05, ** *P* ≤ 0.001, **** *P* ≤ 0.0001.

Neurotransmitters are released at the site of active zones; however, not all active zones are functional. To determine if these active zones are functional, we recorded miniature excitatory junction potentials (mEJPs) at the third instar larval NMJ over two minutes. Consistent with previous research, LoF mutants showed a decrease in the mEJP frequency that was fully rescued when *NLGN3*^*Ref*^ was expressed in a *Nlg3*^*TG4/Null*^ background [40]. Surprisingly, the expression of the *NLGN3*^*R175W*^ and *NLGN3*^*R451C*^ variants fully or partially rescued this phenotype, respectively; however, the expression of the *NLGN3*^*R597W*^ variant failed to rescue this defect (Fig 6I–6J). This effect was consistent in both males and females (S8A–S8B Fig). Additionally, we did not observe any difference in the mEJP amplitude or kinetics in female larvae; however, male *Nlg3*^*TG4/Null*^ larvae showed a decrease in rise time and amplitude which was fully rescued when *NLGN3*^*Ref*^ was expressed in a *Nlg3*^*TG4/Null*^ background (S8 Fig). Together, this suggests that the presence of BRP puncta does not correlate with the ability of the variants to rescue vesicle release. This is likely because not all active zones are fully functional. It is possible that in the p.R175W and p.R451C variants, having an increase in active zones may help restore the threshold needed to maintain the frequency of spontaneous vesicle release.

*Nlg3* has been implicated not only in synapse development but also in synaptic vesicular trafficking [40]. To determine if our missense variants disrupt vesicle trafficking, we used the FM1–43 styryl dye which functions in an activity dependent manner. Similar to previous reports, *Nlg3* LoF mutants had a significant decrease in dye uptake compared to control animals, indicative of an endocytic defect (S9A–S9C Fig) [40]. The expression of human *NLGN3* reference or variant constructs in a *Nlg3*^*TG4/Null*^ background rescued this dye uptake defect (S9D–S9H Fig). This suggests that the expression of *NLGN3* reference and variants are sufficient to restore endocytosis. However, the dye distribution within the bouton appeared abnormal in the variants tested. In both the control and *NLGN3*^*Ref*^ rescue animals, the dye appeared as puncta within the boutons; however, in the LoF mutants and variant rescue animals, the majority of the dye surrounded the border of the boutons suggesting that vesicle size and/or morphology may be altered.

## Discussion

Although *NLGN3* is an established X-linked ASD susceptibility gene, most variants have yet to be functionally characterized. In this study, we selected three *NLGN3* variants and modeled them in *Drosophila*. Consistent with previous findings, loss of *Nlg3* reduced locomotor activity, increased the number of type 1B boutons, and decreased frequency of mEJP and FM1–43 dye uptake [40,47]. Additionally, we observed phenotypes that were not previously reported such as decreases in sleep latency and increases in nighttime sleep duration and BRP puncta (Table 1). Based on humanization strategies, we rescued or nominally improved many of these LoF phenotypes using human *NLGN3*, demonstrating that human NLGN3 can functionally replace the *Drosophila* protein in many biological instances (Table 1). We then examined the functional consequences of three ASD-associated variants. We showed that the *NLGN3*^*R175W*^ variant, a *de novo* variant identified in a heterozygous female with ASD, was also able to rescue male sleep latency, frequency of mEJP, and FM1–43 dye uptake phenotypes (Table 1). However, when we overexpressed this variant with neuronal drivers, these animals had exacerbated sleep phenotypes, and the boutons were significantly overgrown compared to *NLGN3*^*Ref*^ flies (S3 Table). Furthermore, the introduction of this variant did not alter protein expression levels, suggesting that this variant exhibits GoF-like properties. Although our expression and rescue experiments suggest that this variant is a putative GoF allele, we did not further validate whether this variant functions in a true GoF mechanism. Therefore, additional studies that preserve endogenous protein levels are needed to validate a true GoF mechanism. While we predict the *NLGN3*^*R175W*^ variant to have enhanced activity, there are limitations to this interpretation since this missense variant is not conserved between *Drosophila* and humans. In *Drosophila*, the corresponding residue is preceded by a long serine-rich domain which is not present in vertebrates or *C.elegans*, suggesting that this serine-rich domain is specific to *Drosophila* and may have altered structure and function in the fly. Furthermore, the corresponding residue itself is also not conserved between vertebrates and invertebrates. This lack of conservation limits the interpretation of our results since we are unable to determine if this missense variant is a true GoF allele. Although this variant is not conserved, our findings are insightful since we are comparing the effect of this missense variant to that of the human reference. Further studies in a model organism where this variant is conserved could be used to validate these findings. The *NLGN3*^*R451C*^ variant exhibited a complex mechanism and cannot be classified as a simple LoF or GoF allele. When we expressed this variant in a *Nlg3*^*TG4/Null*^ background, it failed to rescue male sleep behaviors and NMJ morphological defects, and the introduction of this variant caused a significant decrease in protein expression, suggesting it has some LoF properties (Table 1). Yet, female sleep behaviors were nominally improved, though not significantly. When we overexpressed this variant, the boutons were significantly overgrown compared to *NLGN3*^*Ref*^, suggesting it also possesses some GoF properties (S3 Table). Overall, our study points towards a complex mechanism that is likely context dependent. Lastly, the *NLGN3*^*R597W*^ variant failed to rescue sleep deficits in both males and females and frequency of mEJP; however, it was able to restore NMJ morphology and FM1–43 dye uptake (Table 1). Overexpression of this variant with neuronal drivers produced minimal phenotypes, and the protein expression was significantly reduced compared to the *NLGN3*^*Ref*^ allele. (S3 Table). The combination of the rescue and overexpression experiments together suggests that the variant produces a complex, context-dependent phenotype most consistent with a LoF. While we cannot formally conclude that this variant acts as a simple LoF allele, there is little evidence to suggest any GoF phenotypes that result from its expression. Therefore, we suggest that this variant acts as a complex LoF allele, with significant context dependency. Together these results suggest that variants in *NLGN3* can produce a spectrum of functional outcomes, and individual variants can contribute to distinct phenotypes.

**Table 1:** Summary of all rescue assays performed.

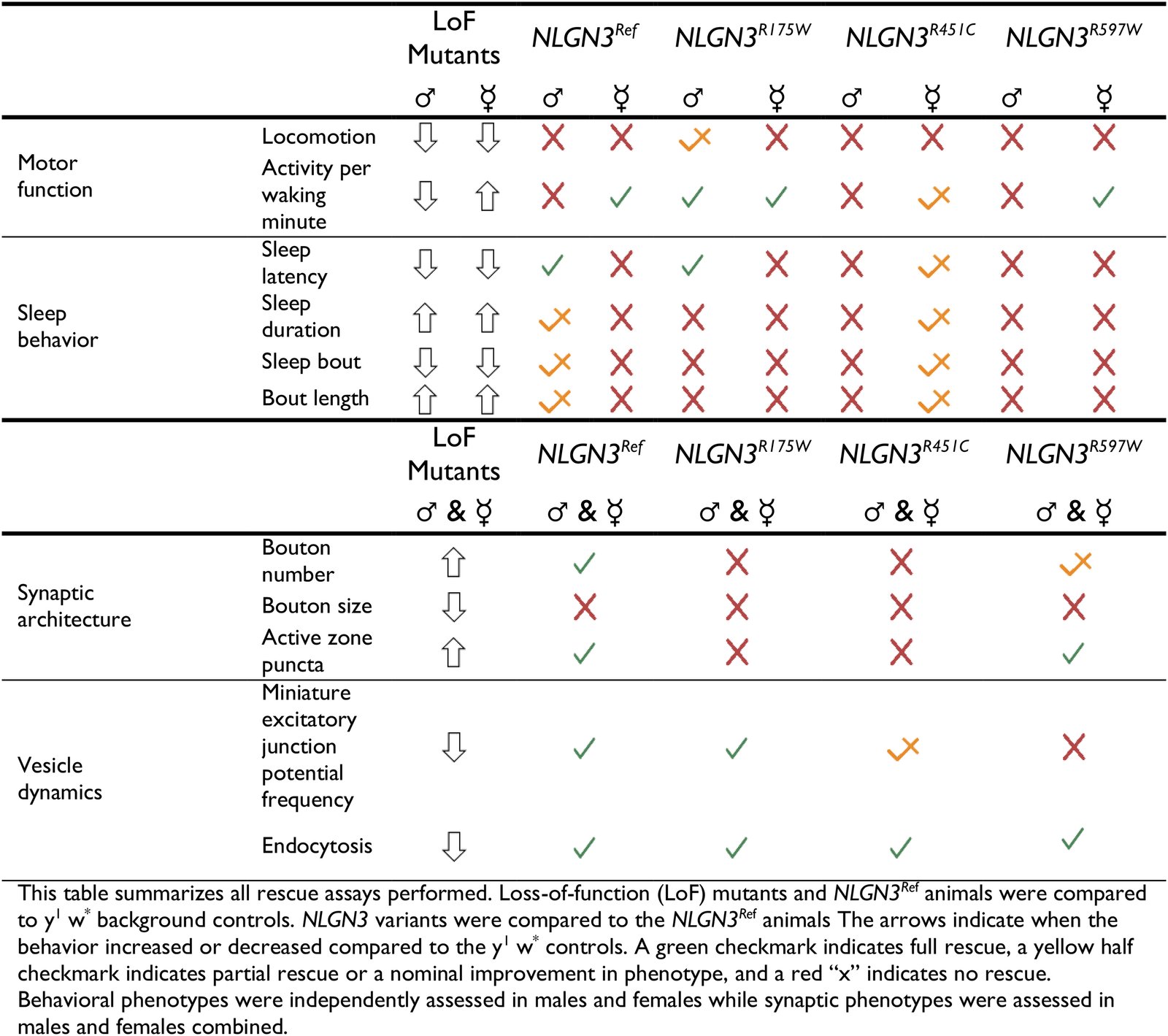

The functional investigation of *NLGN3* variants in ASD has been mostly limited to the *NLGN3*^*R451C*^ allele [11]. Although *NLGN3* variants were originally considered GoF, several early stop gain variants have been identified, suggesting that LoF *NLGN3* variants may also contribute to disease phenotypes [61,62]. Numerous *Nlgn3*^*R451C*^ mouse knock-in studies have supported a causative role in ASD pathophysiology. Several studies have recapitulated ASD-relevant behaviors including deficits in social interaction, hyperactivity, enhanced repetitive motor routines, and surprisingly, enhanced spatial learning abilities [23,27,30]. Consistent with the hypothesis that ASD may alter the excitation/inhibition balance, the *Nlgn*^*R451C*^ variant increases the strength of inhibitory synaptic transmission in the somatosensory cortex and reduces inhibitory synaptic transmission at synapse formed by Parvalbumin basket cells [23,31]. Other studies have shown it increases the strength of the excitatory synaptic transmission in the hippocampus, yet these effects were not observed in *Nlgn3* knockout mice suggesting a GoF mechanism [23,27,31]. Still, others observed an increase in inhibitory synaptic transmission in cholecystokinin positive GABAergic basket cells that was also seen in *Nlgn3* knockout mice suggesting a LoF mechanism [31]. Together, these studies suggest that *NLGN3* may induce cell-type and synapse specific changes. In our study, we observed both LoF and GoF properties for the *NLGN3*^*R451C*^ variant. While *NLGN3*^*R451C*^ failed to rescue male sleep behaviors and NMJ morphological defects, suggesting a LoF phenotype, overexpressing this variant further exacerbated bouton overgrowth, suggesting a GoF phenotype. Our results are consistent with previous studies, further confirming a context-dependent effect. This approach also highlights the importance of utilizing multiple functional assays to understand the pathogenic mechanism of disease since some variants can have distinct effects on different tissue and cell types.

Individuals with ASD are more likely to experience sleep deficits. Roughly 50–80% of individuals with ASD will experience sleep disturbances which is two to three times higher than typical developing children [63]. Sleep disturbances can negatively impact the quality of life not only for the child but also their caregivers [64]. Although the relationship between sleep and ASD is relatively unclear, several studies have implicated *NLGN3* in sleep disruptions [59,60]. *Nlgn3* knockout and *Nlgn3*^*R451C*^ knock-in mice have alterations in spectral power bands during different sleep stages, suggesting that *NLGN3* can affect sleep quality [59,60]. Furthermore, the conditional knockout of *Nlgn3* in medial septum neurons in mice causes hyperactivity of GABAergic neurons and results in social memory impairment and sleep disturbances [65]. While *Drosophila Nlg3* has not been implicated in sleep disruptions prior to our study, sleep abnormalities have been reported in *Drosophila Nlg4* mutants [41]. *Drosophila Nlg4* is highly expressed in the large lateral ventral neurons (l-LNvs) and fan shaped bodies, the sleep centers of the fly brain [41]. Loss of *Nlg4* impaired GABA transmission resulting in hyperactivity and increase sleep latency [41]. Together these studies suggest that the excitatory/inhibitory imbalance may contribute to the pathogenesis of both ASD and sleep disturbances.

In the context of sleep, we were particularly interested in one variant, *NLGN3*^*R597W*^, which was identified in three affected individuals who were diagnosed with ASD and sleep disorders [49,50]. Driven by clinical data, we assessed if *Drosophila Nlg3* may alter sleep behaviors. We identified that loss of *Nlg3* reduced sleep latency, yet this phenotype was rescued in male flies where *NLGN3*^*Ref*^ was expressed, suggesting that *Nlg3* is involved in sleep in flies as well. Furthermore, male flies carrying *NLGN3* variants failed to rescue sleep duration and sleep consolidation phenotypes whereas *NLGN3*^*Ref*^ was able to nominally improve these phenotypes, confirming that our assays are sensitive enough to detect sleep defects in the *NLGN3*^*R597W*^ variant. Furthermore, we also examined sexual dimorphism of sleep behaviors. Virgin females displayed differential behaviors where the *NLGN3*^*R451C*^ variant nominally improved sleep phenotypes; however, the other variants failed to improve sleep phenotypes. We hypothesize this difference is a result of the sexual dimorphism of sleep behaviors in males and females. Additionally, we evaluated ASD-relevant behaviors including male and female grooming and male courtship. While previous studies have identified that loss of *Nlg3* increases the social space between animals, grooming and courtship behaviors have not been assessed [47]. We did not detect substantial behavioral changes in male grooming or wing-extension behaviors; however, females exhibited an increase in grooming behaviors, further implying the sexual dimorphism of these behaviors.

Although we did not explore the trafficking of *NLGN3* variants, MutPred2 predicted that the *NLGN3*^*R451C*^ and *NLGN3*^*R597W*^ variants will likely impair membrane signaling leading to the mislocalization of the protein. This was confirmed in previous *in vitro* studies that identified the *NLGN3*^*R451C*^ and *NLGN3*^*R597W*^ variants are retained in the endoplasmic reticulum (ER) [23,24,49]. Additionally, MutPred2 predicted that the *NLGN3*^*R451C*^ variant could accumulate misfolded proteins which can lead to cellular stress. This was validated using *in vitro* studies that observed an accumulation of NLGN3 proteins in the ER which activated the unfolded protein response (UPR) leading to a 90% reduction in cell surface protein expression [23,24,49]. However, 10% of the protein is able to be trafficked properly leading to some functional NLGN3 [23]. In contrast, the *NLGN3*^*R597W*^ variant is predicted to alter DNA binding and impair proper posttranslational modification in addition to impairing membrane trafficking. *In vitro* assays suggest that this variant is unable to localize properly to the plasma membrane and increases the activation of the UPR [49]. Interestingly, we observed that both the *NLGN3*^*R451C*^ and the *NLGN3*^*R597W*^ variant had a reduction in protein expression levels without alterations in mRNA expression. This suggests that the resulting protein is likely unstable. Therefore, we hypothesize that even in *Drosophila,* these variants could be misfolded and retained in the ER for degradation. However, additional *in vivo* studies on the localization of these variants are needed. These molecular differences may provide insight into how these variants are altering function. Although the cellular trafficking of the *NLGN3*^*R175W*^ variant has yet to be studied, MutPred2 does not predict mislocalization with this variant but rather alterations in binding interactions. In our fly models, we did not observe any differences in protein expression levels, thereby suggesting that this variant does not increase protein degradation. Therefore, the *NLGN3*^*R597W*^ variant may impair synaptic function without altering synapse development; however, other variants may disrupt synaptic architecture by impairing proper binding interactions or by interacting with noncanonical partners [66]. Understanding whether the *NLGN3*^*R175W*^ variant impairs vesicle trafficking or inhibits binding with presynaptic partners can provide mechanistic insight into how different mutations across the protein can differentially affect synapse function.

Although we have developed transgenic toolkits to assess the pathogenicity of missense variants in *Drosophila*, our study does have some limitations. We utilized overexpression and rescue paradigms to determine the functional consequences of *NLGN3* variants. We recognize that our overexpression experiments with *Elav*-GAL4, *Mef2*-GAL4, and D42-GAL4 may not recapitulate endogenous protein levels; therefore, we also utilized the *Nlg3*^*TG4*^ allele to express human *NLGN3* transgenes in a similar spatial and temporal pattern as fly *Nlg3*. Using this paradigm, we observed that the *NLGN3*^*Ref*^ or *Nlg3*^*WT*^ rescue constructs failed to rescue locomotion. Previous studies have already demonstrated that the short isoform of wild-type fly *Nlg3* expressed in neurons is critical for regulating locomotor behavior [45]. To achieve this rescue, the authors expressed fly *Nlg3*^*WT*^ in all *Elav* positive neuronal cells. However, we have demonstrated that *Nlg3* is expressed in a large subset of *Elav* positive neurons, but not all neuronal populations. Therefore, it is possible that our *Nlg3*^*TG4*^ allele does not express our constructs in cell populations that are important for regulating locomotion. Additionally, the *Nlg3*^*TG4*^ may have a second site hit or expression levels may be suboptimal thereby suppressing rescue. Future studies using knock-in strategies can be employed to determine whether gene dosage is important for regulating locomotion. Additionally, we are unable to evaluate the impact of vertebrate specific dosage compensation on our variants. Unlike human *NLGN3* which is located on the X-chromosome, *Drosophila Nlg3* is located on the 3^rd^ chromosome. Due to the differences in chromosomal location, we are unable to generate variants in a hemizygous context. Furthermore, to maintain dosage compensation, male flies will upregulate the expression of genes located on the X-chromosome by two-fold via the male specific lethal (MSL) complex [67]. This process is inverse of dosage compensation in humans, where females will silence one copy of the X-allele. This results in females who are mosaic for the variants. We are unable to model this mosaicism in our system. However, our system is still useful as it can compare differences between variant and reference alleles in a biologically relevant model.

In this study, we assessed synapse morphology at the NMJ. The *Drosophila* NMJ is experimentally more accessible and structurally more simplified compared to the mammalian brain [39]. The stereotyped architecture of the NMJ and its capacity for developmental and functional plasticity, make it a powerful system for probing the synaptic consequences of *NLGN3* variants [39]. In our study, electrophysiological recordings were performed at the muscle. While *NLGN3* is generally considered a postsynaptic membrane protein primarily expressed in neurons, low levels of fly *Nlg3* mRNA have been detected in the muscle tissue, but the corresponding protein was undetectable [40,45]. Therefore, it is unclear whether *NLGN3* is acting as a postsynaptic protein in the muscle, acting as a presynaptic protein in the dendrites of motor neurons, or acting upstream in the central nervous system. We observed that knockdown of fly *Nlg3* using neuronal drivers was sufficient to alter bouton morphology. However, other studies demonstrated that the expression of *Nlg3* in the muscle and not neurons is able to rescue bouton morphology [40]. Thus, it is possible that the expression of *Nlg3* in both the central nervous system and muscle may regulate NMJ development [44]. Further studies are needed to tease apart this circuit.

In summary, we investigated the functional impact of three ASD-associated *NLGN3* variants using *Drosophila*. We identified functional differences between all three variants that were selected. We showed that a *de novo* variant that has not been functionally studied previously possesses GoF-like properties, whereas the *NLGN3*^*R451C*^ and *NLGN3*^*R597W*^ variants are likely more complex than originally proposed. Additionally, we observed functional differences between the two maternally inherited variants where one variant effects synapse development and the other effects synaptic function. Further studies are needed to mechanistically understand how these variants affect the function of the protein. However, this study provides new insight into the functional differences between *NLGN3* variants and how these functional differences may confer a spectrum of phenotypes seen in individuals with *NLGN3* variants.

## Materials/Subjects and methods

### Bioinformatic methods

ASD-associated NLGN3 variants were identified through the Simons Foundation Autism Research Initiative (SFARI) and ClinVar databases [32,33]. To prioritize variants, we utilized in silico prediction models such as gnomAD, SIFT, PolyPhen2, CADD, REVEL, AlphaMissense, MutationTaster, and MutPred2 [53,68–76]. Clustal Omega was used to align multiple sequences obtained from UniProt and the Jalview software was used to visualize sequence alignment [77–80]. MetaDome was used to visualize pathogenic hotspots [52].

### *Drosophila* stocks and husbandry

Fly stocks were maintained at room temperature, unless otherwise specified. Experiments were conducted in a temperature and humidity-controlled incubator at 25°C, 50% humidity, and a 12-hour light/dark cycle. Flies were reared on standard fly food (water, yeast, soy flour, cornmeal, agar, corn syrup, and propionic acid). The Canton-S and *p{UAS-lacZ}* flies were provided by Hugo Bellen (Baylor College of Medicine, Texas, USA) and the *Nlg3*^*Def1*^(*Nlg3*^*Null*^) flies were provided by Anne Simon (University of Western Ontario, Canada). The remaining fly stocks were obtained from Bloomington *Drosophila* stock center (Indiana University, Indiana, USA), Vienna *Drosophila* research center (Vienna, Austria), and Kyoto *Drosophila* stock center (Kyoto Institute of Technology, Kyoto, Japan): *y*^*1*^ M{vas-int.Dm}ZH-2A w^*^; PBac{y[+]-attP-3B}VK00037 (BDSC #24872), *y*^*1*^ w^*^; Mi{Trojan-GAL4.2}Nlg3MI00445-TG4.2 (Nlg3^*TG4*^) (BDSC #76134), w*; P{GawB}D42 (BDSC #8816), *P{GawB}elav[C155]* (BDSC #458), *P{GAL4-Mef2.R}3* (BDSC #27390), w*; P{UAS-RedStinger}4, P{UAS-FLP.D}JD1, P{Ubip63E(FRT.STOP)Stinger}9F6/CyO (G-Trace) (BDSC #28280), *y*^*1*^ sc^*^ *v*^*1*^ *sev*^*21*^ *P{VALIUM20-mCherry.RNAi}attP2* (BDSC #35785), *P{GawB}elav[C155]; P{UAS-Dcr-2.D}2* (BDSC #25750), *P{KK106100}VIE-260B* (VDRC: v100376), *P{KK110531}VIE-260B* (VDRC: v102055), *P{NIG.14597R}2* (NIG-Fly: 14597R-2)

### Human *NLGN3* transgenic line generation

Human *NLGN3* reference clone (BC051715.1 transcript in pDonr221 plasmid) was obtained from the Kenneth Scott human cDNA collection (Department of Molecular and Human Genetics, Baylor College of Medicine). Variant constructs (p.R175W, p.R451C, and p.R597W) were generated using NEBase Changer Q5 site-directed mutagenesis kit (New England Biolabs Inc #E0554S) and verified by Sanger sequencing. Reference and variant coding sequences (CDS) were subcloned into the p.UASg-HA.attB vector using LR clonase enzymes (Thermo Fisher Scientific, #12538120) [81]. Verified plasmids were microinjected into *vas-phiC31; VK0037* embryos [82]. All constructs were injected into the VK37 docking site to avoid positional effects. Transgenic male flies were crossed to *y*^*1*^ w^*^ virgin female flies, and successful integration was confirmed by selecting for the mini white gene.

### RNA extraction and Real-time quantitative PCR

Total RNA was extracted from heads of 20 adult flies per genotype using *Quick*-RNA Miniprep Plus kit (Zymo Research #R1057) followed by cDNA synthesis using iScript cDNA Synthesis kit (Bio-Rad #1708890). cDNA concentrations were normalized followed by quantitative PCR experiments using iQ SYBR Green Supermix (Bio-Rad #1708880) on a CFX Opus 96 Real-Time PCR system (Bio-Rad, California, USA). Gene expression was normalized to the *rp49* gene. Primers were designed with PrimerBlast and purified by high-performance liquid chromatography. Primer sequences are as follows:

*rp49* Fwd 5’-GCTAAGCTGTCGCACAAATG-3’
*rp49* Rev 5’-GTTCGATCCGTAACCGATGT-3’
*Nlg3* Fwd 5’-GCAATATGGAATCGAGGCGG-3’
*Nlg3* Rev 5’-AATCACCGAATCGCGTCTG-3’
*NLGN3* Fwd 5’- CGGGTTGGAGTGCTAGGTTT-3’
*NLGN3* Rev 5’- ATATTCTCGCTCACCCAGCG-3’

### Western blot assay

20 adult heads (10 males and 10 females) per genotype were homogenized in ice cold RIPA buffer (GenDEPOT #R4100-010) with 2X protease inhibitor cocktail (APExBIO #K1010). Once thoroughly homogenized, samples were centrifuged for 20 minutes at 15000 RPM until debris was settled. The supernatant was diluted with equal volume of 2X Laemmli sample buffer (Bio-Rad #1610737) and 10% dithiothreitol (Thermo Fisher Scientific #P2325) and boiled for 10 minutes at 60°C. 15 µL of each sample was loaded into 4–20% tris-glycine precast gels (Bio-Rad #4561095). PVDF membrane was activated by applying 100% methanol for one minute and equilibrated in transfer buffer (1X Tris-Glycine native buffer (GenDEPOT #T8052-050) with 10% methanol) for 15 minutes. Protein was transferred from the gel to a 0.2 µm PDVF membrane using a wet transfer method at 0.2A. Membrane was then blocked in 5% blocking solution for 2 hours (1x TBST with 5.0g nonfat dry milk). Membrane was incubated overnight at 4°C with rabbit anti-NLGN3 (ABCAM #ab244265, 1:500), rabbit anti-Nrx (MyBioSource #MBS9458520, 1:500) and mouse anti-α-Tubulin (Sigma Aldrich #T6199, 1:1000) primary antibodies in blocking solution. The membrane was washed at least three times with 1xTBST then incubated with secondary antibody (goat anti-rabbit IgG (H + L) HRP [Thermo Fisher Scientific #31460, 1:5000] and donkey anti-mouse IgM HRP [Jackson ImmunoResearch #715035140, 1:5000] diluted in blocking solution). Western Lightning Plus-ECL (Revvity #NEL104001EA) was used to develop the membrane. Blots were imaged using a ChemiDoc MP Imaging System (Bio-Rad) and analyzed with ImageJ software.

### Longevity Assay

Eclosed flies were separated by sex and genotype and maintained at 25°C. 10 flies of the same sex and genotype were group housed together. Flies were transferred into a new vial every 3–4 days and survival was determined. A Log-rank Mantel Cox test was used to determine significance.

### *Drosophila* behavior assays

Five-to-seven-day old male or virgin female flies were placed in *Drosophila* Activity Monitors (DAM, Trikinetics, Massachusetts, USA) and housed at 25°C, 50% humidity on a 12-hour light/dark cycle for seven days. Virgin female flies were used since mated females will lay eggs which will interfere with the infrared beam. Inactive periods longer than five minutes were classified as sleep. Total activity and sleep behaviors were analyzed using MATLAB SleepMat software [83]. Sleep latency was defined as time from lights off to sleep onset. Sleep consolidation is composed of the number of sleep bouts and the average sleep bout length.

Locomotor, grooming, and courtship behaviors were performed as previously described [84]. Socially naive males or females were collected by isolating pupae in 16 × 100 polystyrene vials with 1 mL of fly food. Behavior was assessed in a custom made 6 well acrylic [85]. One 6-to-10-day old mutant or rescue male fly and one 6-to-10-day old Canton-S virgin female and vice versa was introduced into the chamber well through aspiration. Behavior was recorded with a Basler 1920UM, 1.9MP, 165FPS, USB3 Monochromatic camera and the BASLER Pylon module, with an adjusted capturer rate of 33 frames per second. Videos were compiled using a custom MATLAB script. Fly positions were tracked with CalTech Flytracker [86]. Machine learning assessment for courtship and grooming behaviors were performed using JAABA [87].

### Brain immunostaining

*Nlg3*^*TG4*^ was crossed with w*; P{UAS-RedStinger}4, P{UAS-FLP.D}JD1, P{Ubip63E(FRT.STOP)Stinger}9F6/CyO to visualize endogenous *Drosophila Nlg3* expression [57]*.* Adult brains were dissected in 1x phosphate buffer saline (PBS) and fixed with 4% paraformaldehyde (PFA) for 20 minutes at room temperature and washed three times in 1% Triton/PBS (1% PBST). Samples were incubated overnight at 4°C with mouse anti-Repo [Developmental Studies Hybridoma Bank (DSHB, University of Iowa): 8D12, 1:200] and rat anti-Elav (DSHB: 7E8A10, 1:200) diluted in PBS/1% Triton/5% normal donkey serum (NDS). Following primary antibody staining, samples were washed three times with 1% PBST and incubated with anti-mouse-405 or anti-rat-405 (Jackson ImmunoResearch #715-475-151 and #712-475-153, 1:200) for two hours at room temperature, then mounted in Vectashield mounting media (Vector Laboratories #H-1000–10). Images were acquired using a Z-stack maximal projection on a LSM710 confocal microscope (Carl Zeiss) with a 10X objective lens. Images were processed using ImageJ software.

### *Drosophila* neuromuscular junction morphology

Muscle wall of third instar larvae (muscle 6/7 of segment A4) and abdominal body wall of 7-day-old adult flies were dissected on sylgard plates in 1X PBS and fixed with 4% PFA for 20 minutes. Samples were washed three times with 1% PBST and incubated overnight at 4°C with primary antibodies diluted in 1% PBST and 5% NDS. After three additional 1% PBST washes, samples were incubated with secondary antibodies for two hours at room temperature, mounted on Vectashield mounting media and imaged using a Z-stack maximal projection on a LSM710 confocal microscope with a 40X oil objective lens. Images were processed using ImageJ software. Primary antibodies: mouse anti-DLG (DSHB: 4F3, 1:50), mouse anti-BRP (DSHB: nc82, 1:25), and mouse anti-CSP (DSHB: 6D6, 1:25). Secondary antibodies: Alexa Fluor 488 AffiniPure Goat Anti-Horseradish Peroxidase (Jackson ImmunoResearch #123-545-021, 1:200), mouse anti-Cy3 (Jackson ImmunoResearch #715-165-151, 1:200), mouse anti-647 (Jackson ImmunoResearch #715-605-151).

### Electrophysiology recordings

Electrophysiological recordings were performed as described [88]. Wandering third instar larvae were dissected and recordings were obtained from muscle 6 of abdominal segments A3 and A4 with HL-3 solution containing 1mM Ca^2+^ (7.2 pH). HL-3 solution consisted of 70 mM NaCl, 5 mM KCl, 20 mM MgCl_2_, 10 mM NaHCO_3_, 5 mM trehalose, 115 mM sucralose, and 5 mM HEPES. Microelectrodes (10–20 MΩ) made with borosilicate glass were pulled using a micropipette puller (P-97; Sutter Instrument, California, USA) and filled with 3M KCl. Spontaneous miniature excitatory junction potentials (mEJPs) were recorded with an Axoclamp 900A amplifier (Molecular Devices, California, USA) in bridge mode, digitized with Digidata 1550 digitizer (Molecular Devices), collected with pClamp10.4 software (Molecular Devices), and analyzed using AxoGraph X software. mEJPs were recorded over a period of 120 seconds. Only cells with a resting potential between –50 to –80 mV and an input resistance > 4mΩ were used for analysis. All intracellular recordings were performed at room temperature.

### FM1–43 uptake assay

FM1–43 dye uptake assays were performed as previously described [89]. Wandering third instar larval body walls (muscle 6/7 of segment A4) were dissected on Sylgard plates in Ca^2+^ free HL-3 solution. To stimulate endocytosis, samples were incubated with 10 μm FM1–43 dye in HL-3 solution containing 90mM KCl and 1.5mM Ca^2+^ for 5 minutes then washed five times with Ca^2+^ free HL-3 solution. Imaging was performed on a LSM800 Airyscan confocal microscope (Carl Zeiss) with a 63x water objective lens and analyzed using ImageJ.

### Statistical analysis

Statistical analysis was performed in Prism v10.6 (GraphPad). Data was tested for normality and variance. Normally distributed data where equal variance was violated were analyzed using Welches ANOVA followed by Dunnet’s T3 multiple comparison test, normally distributed data with equal variance was analyzed using one-way ANOVA followed by Sidak’s multiple comparison test, and non-normal data used Kruskal-Wallis with Dunn’s test. *P* < 0.05 was considered significant. All data is visualized as a bar chart (mean ± SEM, including individual points). Sample sizes are described in the figure legends.

## Supporting information

S1 Table Identified 23 ASD-associated *NLGN3* missense variants and used bioinformatic programs and clinical data to categorize the variants. This table summarizes all ASD-associated *NLGN3* variants published in SFARI or ClinVar databases with the year the variant was submitted. Variants selected for functional analysis are highlighted in gray. The recurrence of a variant within a family are noted in the parentheses by the biological sex. Allele frequency was obtained from gnomAD. CADD: combined annotation dependent depletion; PolyPhen2: polymorphism phenotyping 2; SIFT: sorting intolerant from tolerant, REVEL: rare exome variant ensemble learner. ^1^Clinical phenotypes were obtained using human phenotype ontology terms.
(DOCX)

S2 Table Summary of *in silico* prediction tools used to determine the pathogenic effect of three ASD-associated *NLGN3* variants. This table summarizes the prediction tools used to determine the pathogenicity of each variant. SIFT: sorting intolerant from tolerant; PolyPhen2: polymorphism phenotyping 2; CADD: combined annotation dependent depletion; REVEL: rare exome variant ensemble learner; MutPred2: mutation predictor 2; ADP: adenosine diphosphate ribosylation. CADD and REVEL prediction tools combine information from multiple tools.
(DOCX)

S3 Table Summary of overexpression assays performed. This table summarizes overexpression assays performed. *NLGN3*^*Ref*^ animals were compared to *LacZ* animals. *NLGN3* variants were compared to *NLGN3*^*Ref*^ animals. Arrows indicate whether the behavior increased or decreased from their respective controls. ns = no statistical difference when compared to their respective controls.
(DOCX)

S1 Data Raw data. Numerical data used for all graphs including statistical tests and summary statistics.
(XLSX)

S1 Fig Conservation of all ASD-associated *NLGN3* missense variants. Evolutionary conservation of published ASD-associated *NLGN3* missense variants. Human (NM_018977.4), mouse (Q8BYM5), chicken (A0A8V0Z5J3), zebrafish (D0EVX1), *Drosophila* (Q9VIC6), and *C. elegans* (Q9XTG1) were used. Variants with an asterisk were chosen for functional characterization.
(TIF)

S2 Fig *NLGN3* variants are well conserved across other *NLGN* family members. A) Evolutionary conservation of amino acids across other human NLGN family members. B, E) *Nlg3*^*TG4*^ driving a G-trace reporter line was used to identify the developmental expression of *Nlg3*. C) anti-Elav staining labels neurons. D) Merging the expression of *Nlg3* and Elav-positive cells identifies that *Nlg3* is expressed in neurons during development. F) anti-Repo staining labels glial cells. G) Merging *Nlg3* and Repo-positive cells shows some overlap between *Nlg3* and glia during development. Scale bar: 100 μm.
(TIF)

S3 Fig *NLGN3* variants have altered behavioral phenotypes. A) Quantification of the activity per waking minute (n > 35 flies), locomotion (n > 16 flies), and grooming (n > 16 flies) behaviors in adult virgin females (Kruskal Wallis with Dunn’s multiple comparisons test, ns *P* > 0.05, * *P* ≤ 0.05, ** *P* ≤ 0.01, **** *P* ≤ 0.0001). B) Quantification of the activity per waking minute (n > 19 flies; Welch’s ANOVA with Dunnett’s T3 multiple comparison test ns *P* > 0.05, * *P* ≤ 0.05, **** *P* ≤ 0.0001), locomotion, grooming, and courtship behaviors in adult males (n > 16 flies; Kruskal Wallis with Dunn’s multiple comparisons test, ns *P* > 0.05, * *P* ≤ 0.05, ** *P* ≤ 0.01, *** *P* ≤ 0.001,**** *P* ≤ 0.0001).
(TIF)

S4 Fig Overexpression of the p.R175W variant alters sleep behaviors in males. A) Quantification of total activity in *NLGN3* overexpression models using a neuronal GAL4 driver (n ≥ 30 flies). B) Quantification of activity per waking minute in *NLGN3* overexpression models (n ≥ 30 flies). C) Quantification of sleep duration during lights on (n ≥ 30 flies, Welch’s ANOVA with Dunnett’s T3 multiple comparison test, ** *P* ≤ 0.01, **** *P* ≤ 0.0001). D) Quantification of sleep duration during lights off (n ≥ 30 flies). E) Quantification of sleep latency (n ≥ 30 flies). F) Quantification of average sleep bout number during lights off (n ≥ 30 flies, Welch’s ANOVA with Dunnett’s T3 multiple comparison test, ns *P* > 0.05, *** *P* ≤ 0.0001). G) Quantification of average sleep bout length during lights off (n ≥ 30 flies). Kruskal Wallis with Dunn’s multiple comparison test, for A,B,D,E,G; ns *P* > 0.05, * *P* ≤ 0.05, ** *P* ≤ 0.01, *** *P* ≤ 0.001, **** *P* ≤ 0.0001.
(TIF)

S5 Fig *NLGN3* variants do not impair branching. A) Quantification of the number of branches in larval neuromuscular junctions at muscle 6/7 of abdominal segment A4 from *y*^*1*^ w^*^, Nlg3^*TG4/Null*^*, Nlg3*^*Null/Null*^*, NLGN3*^*Ref*^*; Nlg3*^*TG4/Null*^*, NLGN3*^*R175W*^*; Nlg3*^*TG4/Null*^*, NLGN3*^*R451C*^*; Nlg3*^*TG4/Null*^, and *NLGN3*^*R597W*^*; Nlg3*^*TG4/Null*^ larvae (n ≥ 22 larvae, Kruskal-Wallis test; *P* = 0.0929). B) Quantification of the number of boutons in larval neuromuscular junctions at muscle 6/7 of abdominal segment A4 in *y*^*1*^ w^*^, Nlg3^*TG4/Null*^*, NLGN3*^*Ref*^*; Nlg3*^*TG4/Null*^, and *Nlg3*^*WT*^*; Nlg3*^*TG4/Null*^ (n > 25 larvae; Welch’s ANOVA with Dunnett’s T3 multiple comparisons test ns *P* > 0.05, * *P* ≤ 0.05, ** *P* ≤ 0.01, *** *P* ≤ 0.001). C) Quantification of bouton size (n > 25 larvae; Welch’s ANOVA with Dunnett’s T3 multiple comparisons test ns *P* > 0.05, * *P* ≤ 0.05, **** *P* ≤ 0.0001).
(TIF)

S6 Fig Overexpressing *NLGN3* variants with neuronal drivers alter synapse morphology. A) Representative images of type 1B boutons at muscle 6/7 of abdominal segment A4 from overexpressing *LacZ*, *NLGN3*^*Ref*^*, NLGN3*^*R175W*^*, NLGN3*^*R451C*^, and *NLGN3*^*R597W*^ using a *Nlg3*^*TG4*^ driver. B) Quantification of bouton number (n ≥ 16 larvae). C) Representative images of type 1B boutons at muscle 6/7 of abdominal segment A4 from overexpressing *LacZ*, *NLGN3*^*Ref*^*, NLGN3*^*R175W*^*, NLGN3*^*R451C*^, and *NLGN3*^*R597W*^ using a neuronal driver (*Elav*-GAL4). D) Quantification of bouton number (n ≥ 17 larvae). E) Representative images of type 1B boutons at muscle 6/7 of abdominal segment A4 from overexpressing *LacZ*, *NLGN3*^*Ref*^*, NLGN3*^*R175W*^*, NLGN3*^*R451C*^, and *NLGN3*^*R597W*^ using a motor neuron (D42-GAL4) driver. F) Quantification of bouton number (n ≥ 20 larvae). G) Representative images of type 1B boutons at muscle 6/7 of abdominal segment A4 from overexpressing *LacZ*, *NLGN3*^*Ref*^*, NLGN3*^*R175W*^*, NLGN3*^*R451C*^, and *NLGN3*^*R597W*^ using a muscle driver (*Mef2*-GAL4). H) Quantification of bouton number (n ≥ 19 larvae). One-way ANOVA with Sidak’s multiple comparison test, ns *P* > 0.05,* *P* ≤ 0.05, ** *P* ≤ 0.01, **** *P* ≤ 0.0001.
(TIF)

S7 Fig Synaptic architecture is dysregulated post development. A-G) Representative images of 7-day-old adult abdominal neuromuscular junctions (NMJ) from *y*^*1*^ w^*^, Nlg3^*TG4/Null*^*, Nlg3*^*Null/Null*^*, NLGN3*^*Ref*^*; Nlg3*^*TG4/Null*^*, NLGN3*^*R175W*^*; Nlg3*^*TG4/Null*^*, NLGN3*^*R451C*^*; Nlg3*^*TG4/Null*^, and *NLGN3*^*R597W*^*; Nlg3*^*TG4/Null*^ flies stained with anti-CSP, a presynaptic marker. Scale bar: 10 μm. H) Quantification of bouton number in an adult NMJ (n ≥ 20 flies). One-way ANOVA with Sidak’s multiple comparison test; ns *P* > 0.05, ** *P* ≤ 0.01, *** *P* ≤ 0.001, **** *P* ≤ 0.0001.
(TIF)

S8 Fig *NLGN3* variants do not alter mEJP kinetics. A) Quantification of the frequency (n > 7 larvae; Welch’s ANOVA with Dunnett’s T3 multiple comparisons test), rise time (n > 7 larvae; Welch’s ANOVA with Dunnett’s T3 multiple comparisons test), decay time (n > 6 larvae; One-way ANOVA with Sidak’s multiple comparisons test), and amplitude (n > 7 larvae; One-way ANOVA with Sidak’s multiple comparisons test) of miniature excitatory junction potentials (mEJP) in female *y*^*1*^ w^*^, Nlg3^*TG4/Null*^*, Nlg3*^*Null/Null*^*, NLGN3*^*Ref*^*; Nlg3*^*TG4/Null*^*, NLGN3*^*R175W*^*; Nlg3*^*TG4/Null*^*, NLGN3*^*R451C*^*; Nlg3*^*TG4/Null*^, and *NLGN3*^*R597W*^*; Nlg3*^*TG4/Null*^ larvae. ns *P* > 0.05, * *P* ≤ 0.05, *** *P* ≤ 0.001, **** *P* ≤ 0.0001. B) Quantification of the frequency (n > 8 larvae; One-way ANOVA with Sidak’s multiple comparisons test), rise time (n > 8 larvae; Welch’s ANOVA with Dunnett’s T3 multiple comparisons test), decay time (n > 5 larvae; Kruskal Wallis with Dunn’s multiple comparisons test), and amplitude (n > 7 larvae; One-way ANOVA with Sidak’s multiple comparisons test) of mEJP in male *y*^*1*^ w^*^, Nlg3^*TG4/Null*^*, Nlg3*^*Null/Null*^*, NLGN3*^*Ref*^*; Nlg3*^*TG4/Null*^*, NLGN3*^*R175W*^*; Nlg3*^*TG4/Null*^*, NLGN3*^*R451C*^*; Nlg3*^*TG4/Null*^, and *NLGN3*^*R597W*^*; Nlg3*^*TG4/Null*^ larvae. ns *P* > 0.05, * *P* ≤ 0.05, ** *P* ≤ 0.01, *** *P* ≤ 0.001, **** *P* ≤ 0.0001. C). Representative mEJP amplitude traces from muscle 6 at abdominal segments A3 and A4 from *y*^*1*^ w^*^, Nlg3^*TG4/Null*^*, Nlg3*^*Null/Null*^*, NLGN3*^*Ref*^*; Nlg3*^*TG4/Null*^*, NLGN3*^*R175W*^*; Nlg3*^*TG4/Null*^*, NLGN3*^*R451C*^*; Nlg3*^*TG4/Null*^, and *NLGN3*^*R597W*^*; Nlg3*^*TG4/Null*^ larvae.
(TIF)

S9 Fig *NLGN3* rescues endocytic defects. A-F) Representative images of boutons from *y*^*1*^ w^*^, Nlg3^*TG4/Null*^*, Nlg3*^*Null/Null*^*, NLGN3*^*Ref*^*; Nlg3*^*TG4/Null*^*, NLGN3*^*R175W*^*; Nlg3*^*TG4/Null*^*, NLGN3*^*R451C*^*; Nlg3*^*TG4/Null*^, and *NLGN3*^*R597W*^*; Nlg3*^*TG4/Null*^ larvae loaded with FM1–43 dye. H) Quantification of the FM1–43 dye uptake measured by the intensity of the dye normalized to the size of the boutons (N ≥ 6 larvae, n = 5 boutons per larva), One-way ANOVA with Sidak’s multiple comparison test; ns *P* > 0.05, * *P* ≤ 0.05, *** *P* ≤ 0.001. Scale bar: 10 μm.
(TIF)

S1 RawGel Raw uncropped western blot images. Blots were stained with human NLGN3 (ab244265), *Drosophila* α-Tubulin, and *Drosophila* Neurexin (MBS9458520).
(TIF)
