# Supplementary material for: NLGN3 autism variants have distinct functional impact on synapses and sleep behavior in Drosophila
Source: PLoS Genet. 2026 Oct 16;22(10):e1012341. doi: 10.1371/journal.pgen.1012341 (PMC13705613; doi:10.1371/journal.pgen.1012341)
Supplement: Supplementary file 1 [file pgen.1012341.s001.docx]

# S1 Table: Identified 23 ASD-associated *NLGN3* missense variants and used bioinformatic programs and clinical data to categorize the variants

|  | Variant (NM_018977.4) | Chromosome location | cDNA | Conservation (vertebrates, invertebrates, both) | Inheritance Pattern | Biological Sex | Allele Frequency | CADD | PolyPhen2 | SIFT | REVEL | AlphaMissense | AlphaMissense Classification | Multigenic Cause? | Clinical Phenotype | Additional neurological comorbidities (Y/N) | Sleep Disturbances (Y/N) | Sufficient Clinical Data (Y/N) | Publication |
| --- | --- | --- | --- | --- | --- | --- | --- | --- | --- | --- | --- | --- | --- | --- | --- | --- | --- | --- | --- |
| *de novo* variants | p.R175W | X:71155219 | c.523C>T | Vertebrates | *De* *Novo* | Female | 1.65E-05 | 30 | 0.982 | 0 | 0.477 | 0.5203 | Ambiguous | N/A | ASD, ID | Y | N | N | Iossifov et al. (2014) |
|  | p.G406S | X:71167373 | c.1216G>A | Vertebrates | *De Novo* | Female | 8.25E-07 | 26.7 | 0.023 | 0.01 | 0.295 | 0.082 | Likely Benign | N/A | Delayed language development, epilepsy, behavioral abnormalities, ASD | Y | N | Y | Xu et al. (2014) |
|  | p.R640C | X:71169528 | c.1918C>T | None | *De Novo* | Male | 3.30E-06 | 25.9 | 0.761 | 0 | 0.382 | 0.3201 | Ambiguous | N/A | ASD | N | N | N | ClinVar (2024) |
|  | p.L721R | X:71169772 | c.2162T>G | None | *De Novo* | Male | Absent | 17.95 | 0.063 | 0.19 | 0.116 | 0.0665 | Likely Benign | N/A | ASD, developmental delay, speech delay, ID, regression, repetitive movement, self harm, echolalia, | Y | N | Y | Sheth et al. (2023) |
| Maternally inherited variants | p.R451C | X:71167508 | c.1351C>T | Both | Maternal | Males (x2) | Absent | 33 | 1 | 0 | 0.767 | 0.9417 | Likely Pathogenic | N/A | ASD, ID, clonic-tonic generalized seizures, | Y | N | Y | Jamain et al. (2003) |
|  | p.T542I | X:71167782 | c.1625C>T | Vertebrates | Maternal | Male | Absent | 31 | 0.962 | 0 | 0.834 | 0.9838 | Likely Pathogenic | N/A | Motor delay, obesity, microcephaly, severe global developmental delay, absent speech, ASD | Y | N | Y | ClinVar (2024) |
|  | p.I252F | X:71164229 | c.754A>T | Vertebrates | Maternal | Male | Absent | 28.5 | 0.946 | 0 | 0.835 | 0.8807 | Likely Pathogenic | N/A | Overgrowth, obesity, hemihypertrophy, short stature, failure to thrive, decreased response to growth hormone stimulation test, elevated circulating growth hormone concentration, tall stature, autistic behavior, abnormal large intestine  morphology | N | N | Y | ClinVar (2022) |
|  | p.D594N | X:71169390 | c.1780G>A | Vertebrates | Maternal | Male | Absent | 27.5 | 0.793 | 0 | 0.379 | 0.5297 | Ambiguous | N/A | ASD, Lennox - Gastaut syndrome, abnormal psychomotor development, ID, dysmorphic  features | Y | N | Y | Sedlackova et al. (2024) |
|  | p.M538V | X:71167769 | c.1612A>G | Both | Maternal | Male (x3) | Absent | 27 | 0.998 | 0 | 0.713 | 0.8953 | Likely Pathogenic | N/A | ASD | N | N | N | ClinVar (2021) |
|  | p.V341A | X:71167179 | c.1022T>C | Vertebrates | Maternal | Male | 2.31E-05 | 26.1 | 0.698 | 0 | 0.437 | 0.5143 | Ambiguous | N/A | ASD, ID, hyperactive, mild hearing loss, obesity, occipital frontal circumference at 65th percentile, dysmorphic feature | Y | N | Y | Yu et al. (2013) |
|  | p.P514S | X:71167697 | c.1540C>T | Both | Maternal | Males (x2) | Absent | 26 | 0.97 | 0 | 0.81 | 0.9865 | Likely Pathogenic | N/A | ASD, ID, speech regression | Y | N | Y | Quartier et al. (2019) |
|  | p.R777W | X:71169939 | c.2329C>T | Vertebrates | Maternal | Male | Absent | 24.8 | 1 | 0 | 0.756 | 0.6974 | Likely Pathogenic | N/A | ASD | N | N | N | ClinVar (2023) |
|  | p.S249N | X:71164221 | c.746G>A | Vertebrates | Maternal | Male | Absent | 24.2 | 0.001 | 0.03 | 0.144 | 0.1924 | Likely Benign | N/A | ASD, ID | Y | N | N | ClinVar (2021) |
|  | p.V326M | X:71167133 | c.976G>A | None | Maternal | Male | 3.31E-06 | 24.2 | 0.102 | 0.05 | 0.119 | 0.0821 | Likely Benign | EHMT1 c.3295C>G | ASD | N | N | N | Jiang et al. (2013) |
|  | p.T632A | X:71169504 | c.1894A>G | Vertebrates | Maternal | Males (x2) | 3.83E-03 | 20.7 | 0 | 0.08 | 0.125 | 0.0605 | Likely Benign | N/A | ASD | N | N | N | Blasi et al. (2006) |
| Sleep associated variants | p.R597W | X:71169399 | c.1789C>T | Both | Maternal | Males (x3) | 8.29E-07 | 28.8 | 1 | 0 | 0.789 | 0.9942 | Likely Pathogenic | N/A | ASD, ID, sleep disorders, mild microcephaly, absence of speech | Y | Y | Y | Redin et al. (2014), Quartier et al. (2019) |
|  | p.P120L | X:71148108 | c.359C>T | Vertebrates | De Novo | Male | 8.26E-07 | 28.7 | 0.802 | 0 | 0.712 | 0.9715 | Likely Pathogenic | N/A | Retrognathia, hypertelorism, prominent antihelix, up slanted palpebral fissure, behavioral abnormality, ASD, impaired social interactions, self-mutilation, delayed speech and language development, global developmental delay, motor delay, joint hypermobility, abnormality of abdomen morphology, sleep disturbance, hyperlordosis, thick nasal alae, short 5th metacarpal, intellectual disability^1^ | Y | Y | Y | Laurie et al (2025) |
| Variants with unknown inheritance or biological sex | p.G188R | X:71155258 | c.562G>A | Both | Maternal | Unknown | Absent | 33 | 0.995 | 0 | 0.949 | 0.9959 | Likely Pathogenic | N/A | ASD | N | N | N | ClinVar (2025) |
|  | p.E139K | X:71148164 | c.415G>A | Both | Unknown | Male | 8.26E-07 | 32 | 1 | 0.02 | 0.87 | 0.9869 | Likely Pathogenic | N/A | ASD | N | N | N | ClinVar (2025) |
|  | p.A442T | X:71167481 | c.1324G>A | Vertebrates | Unknown | Unknown | Absent | 32 | 0.958 | 0 | 0.408 | 0.8986 | Likely Pathogenic | N/A | ASD | N | N | N | ClinVar (2022) |
|  | p.Y147H | X:71148188 | c.439T>C | Both | Unknown | Male | Absent | 28.3 | 1 | 0 | 0.839 | 0.9762 | Likely Pathogenic | ASH1L c.3987A>G | ASD, motor coordination disorder | N | N | N | Greco et al. (2025) |
|  | p.N216S | X:71155343 | c.647A>G | Both | Unknown | Unknown | Absent | 25 | 0.965 | 0 | 0.588 | 0.5246 | Ambiguous | N/A | ASD | N | N | N | ClinVar (2021) |
|  | p.H761R | X:71169892 | c.2282A>G | None | Unknown | Unknown | 7.52E-06 | 18.56 | 0.28 | 0.02 | 0.167 | 0.0728 | Likely Benign | N/A | ASD | N | N | N | ClinVar (2024) |

This table summarizes all ASD-associated *NLGN3* variants published in SFARI or ClinVar databases with the year the variant was submitted. Variants selected for functional analysis are highlighted in gray. The recurrence of a variant within a family are noted in the parentheses by the biological sex. Allele frequency was obtained from gnomAD. CADD: combined annotation dependent depletion; PolyPhen2: polymorphism phenotyping 2; SIFT: sorting intolerant from tolerant, REVEL: rare exome variant ensemble learner

^1^Clinical phenotypes were obtained using human phenotype ontology terms
