# Supplementary material for: NLGN3 autism variants have distinct functional impact on synapses and sleep behavior in Drosophila
Source: PLoS Genet. 2026 Oct 16;22(10):e1012341. doi: 10.1371/journal.pgen.1012341 (PMC13705613; doi:10.1371/journal.pgen.1012341)
Supplement: Supplementary file 2 [file pgen.1012341.s002.docx]

| **S2 Table: Summary of *in silico* prediction tools used to determine the pathogenic effect of three ASD-associated *NLGN3* variants** | | | | |
| --- | --- | --- | --- | --- |
| **Prediction Tools** | **Variant 1** | **Variant 2** | | **Variant 3** |
| cDNA  (NM_018977.4 transcript) | c.523 C>T | c.1351 C>T | | c.1789 C>T |
| Protein | p.R175W | | p.R451C | p.R597W |
| Inheritance Pattern | *De Novo* | Maternally Inherited | | Maternally Inherited |
| Allele Frequency | 1.65E-05 | Absent | | 8.29E-07 |
| SIFT | Deleterious (0) | Deleterious (0) | | Deleterious (0) |
| PolyPhen2 | Probably damaging (0.982) | Probably damaging (1) | | Probably damaging (1) |
| CADD | 30 | 33 | | 28.8 |
| REVEL | 0.477 | 0.767 | | 0.789 |
| AlphaMissense | Ambiguous (0.5203) | Likely pathogenic (0.9417) | | Likely pathogenic (0.9942) |
| MutationTaster | Deleterious | Deleterious | | Deleterious |
| MutPred2 Score | 0.798 | 0.908 | | 0.867 |
| MutPred2 Molecular Mechanism | Loss of intrinsic disorder, loss of B-factor, loss of ADP-ribosylation, gain of proteolytic cleavage | Altered ordered interface, altered transmembrane protein, loss of ubiquitylation | | Altered ordered interface, loss of relative solvent accessibility, altered DNA binding, altered transmembrane protein |
| This table summarizes the prediction tools used to determine the pathogenicity of each variant. SIFT: sorting intolerant from tolerant; PolyPhen2: polymorphism phenotyping 2; CADD: combined annotation dependent depletion; REVEL: rare exome variant ensemble learner; MutPred2: mutation predictor 2; ADP: adenosine diphosphate ribosylation. CADD and REVEL prediction tools combine information from multiple tools. | | | | |
