# Supplementary material for: NLGN3 autism variants have distinct functional impact on synapses and sleep behavior in Drosophila
Source: PLoS Genet. 2026 Oct 16;22(10):e1012341. doi: 10.1371/journal.pgen.1012341 (PMC13705613; doi:10.1371/journal.pgen.1012341)
Supplement: Supplementary file 3 [file pgen.1012341.s003.docx]

| **S3 Table: Summary of overexpression assays performed** | | | | | |
| --- | --- | --- | --- | --- | --- |
|  | | ***NLGN3^Ref^*** | ***NLGN3^R175W^*** | ***NLGN3^R451C^*** | ***NLGN3^R597W^*** |
| **Motor function**  **(Neuronal Driver)** | Locomotion |  |  | ns |  |
|  | Activity per waking minute | ns | ns | ns | ns |
| **Sleep behavior**  **(Neuronal Driver)** | Sleep latency |  | ns | ns | ns |
|  | Sleep duration |  |  | ns |  |
|  | Sleep bout |  |  | ns | ns |
|  | Bout length |  |  | ns | ns |
| **Synaptic architecture** | Bouton number (*Nlg3^TG4^* driver) | ns |  |  | ns |
|  | Bouton number (neuronal driver) | ns |  |  | ns |
|  | Bouton number (motor neuron driver) | ns |  |  | ns |
|  | Bouton number (muscle driver) |  | ns | ns | ns |
| This table summarizes overexpression assays performed. *NLGN3^Ref^* animals were compared to *LacZ* animals. *NLGN3* variants were compared to *NLGN3^Ref^* animals. Arrows indicate whether the behavior increased or decreased from their respective controls. ns = no statistical difference when compared to their respective controls | | | | | |
